# Bolstered bone regeneration by multiscale customized magnesium scaffolds with hierarchical structures and tempered degradation

**DOI:** 10.1016/j.bioactmat.2024.12.002

**Published:** 2025-01-03

**Authors:** Zehui Lv, Bo Peng, Yu Ye, Haojing Xu, Xuejie Cai, Jinge Liu, Jiabao Dai, Yixin Bian, Peng Wen, Xisheng Weng

**Affiliations:** a Department of Orthopedic Surgery, State Key Laboratory of Complex Severe and Rare Diseases, Peking Union Medical College Hospital, Chinese Academy of Medical Science and Peking Union Medical College, Beijing 100730, China; b State Key Laboratory of Clean and Efficient Turbomachinery Power Equipment, Department of Mechanical Engineering, Tsinghua University, Beijing, 100084, China; c Hefei Boya Maite Biomaterials Co., Ltd, Hefei 230000, China

**Keywords:** Hierarchical structure, Tempered degradation, Mg scaffold, 3D printing, Biodegradable bone implants

## Abstract

Addressing irregular bone defects is a formidable clinical challenge, as traditional scaffolds frequently fail to meet the complex requirements of bone regeneration, resulting in suboptimal healing. This study introduces a novel 3D-printed magnesium scaffold with hierarchical structure (macro-, meso-, and nano-scales) and tempered degradation (microscale), intricately customized at multiple scales to bolster bone regeneration according to patient-specific needs. For the hierarchical structure, at the macroscale, it can feature anatomic geometries for seamless integration with the bone defect; The mesoscale pores are devised with optimized curvature and size, providing an adequate mechanical response as well as promoting cellular proliferation and vascularization, essential for natural bone mimicry; The nanoscale textured surface is enriched with a layered double hydroxide membrane, augmenting bioactivity and osteointegration. Moreover, microscale enhancements involve a dual-layer coating of high-temperature oxidized film and hydrotalcite, offering a robust shield against fast degradation. Eventually, this scaffold demonstrates superior geometrical characteristics, load-bearing capacity, and degradation performance, significantly outperforming traditional scaffolds based on in vitro and in vivo assessments, marking a breakthrough in repairing customized bone defects.

## Highlights

• This research presents a novel biomaterial solution for bone regeneration, specifically targeting irregular bone defects. We introduce a magnesium (Mg) scaffold with a hierarchical structure and controlled degradation, engineered to meet the biological, physical, and chemical requirements for effective bone repair. This multiscale approach represents a significant advancement in bone regeneration technology by combining precision, functionality, and degradability in a single scaffold. Our findings offer a promising step forward in the development of customizable bone repair solutions, moving us closer to realizing more effective treatments for complex bone injuries.

## Graphical abstract

**Figure:**
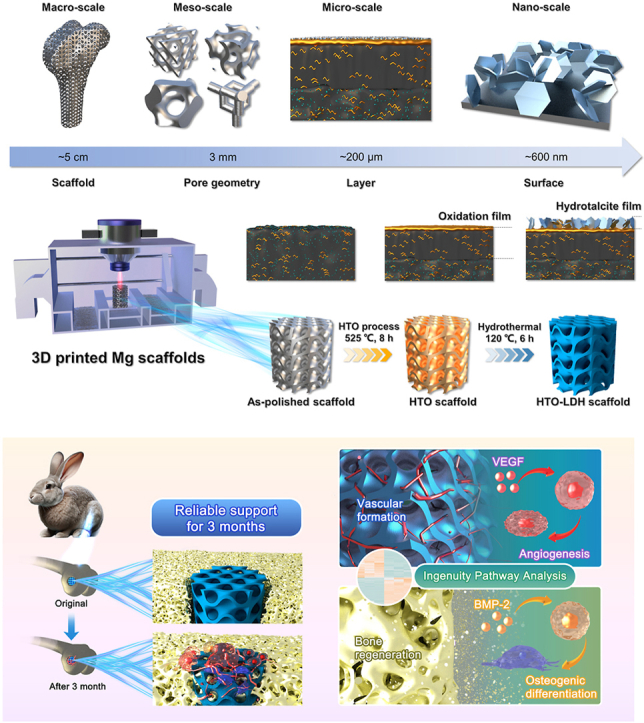

## 1 Introduction

The treatment of large segmental bone defects caused by trauma or tumor resection, in which bone grafting is necessitated, remains a significant clinical challenge, despite the bone being one of the few regenerative tissues with remarkable self-repair capacity [[1], [2], [3], [4]]. Autologous transplantation is currently considered the gold standard for bone grafting, however, the second surgery, the limited availability, and the anatomical mismatch constrain its widespread application [5]. With the development of tissue engineering, synthetic scaffolds have emerged as a promising alternative [[6], [7], [8]]. Achieving a speedy and complete bone recovery depends on two key factors of a scaffold: material and structure [7,9,10]. The ideal scaffold materials should provide adequate mechanical support and osteogenic functions at the early phase of bone regeneration, then gradually degrade, and leave no implant residue after they fulfill their role in aiding bone healing [9,11,12]. While certain artificial bone materials, such as polymers, hydrogels, and ceramics, can fill defects and prove bioactive to promote bone formation, their scaffolds often lack sustained mechanical strength [10]. Metal scaffolds, however, are unfading stars in bone repair materials, owing to their exceptional mechanical properties and biological regulatory functions [13,14].

Magnesium (Mg) alloys, known for their biocompatibility, biodegradability, and excellent mechanical properties, are particularly noteworthy. Mg is an essential life element and can degrade safely in human body. The density and Young's modulus of pure Mg is approximately 1.74 g/cm^3^ and 40 GPa, while the strength of Mg alloy can reach over 300 MPa [15]. These features are roughly comparable to those of the compact bone. Moreover, Mg plays a crucial role in bone metabolism, such as promoting cell proliferation and differentiation, accelerating osseointegration, and new bone formation around implants [[16], [17], [18], [19]]. Thus, Mg alloys hold tremendous potential in the field of biodegradable bone implant devices. Among them, WE43 alloy is one of the limited types that has achieved clinical approval. However, the key challenges with Mg alloys lie in their rapid degradation, which leads to premature structural collapse and the resultant excessive release of hydrogen gas and Mg ions at the local site of implantation [20]. For Mg bone implant scaffolds, a degradation rate of 0.1–0.5 mm/year is generally considered favorable for maintaining the balance between the bone regeneration microenvironment and mechanical integrity ^11^. Many approaches have been developed to inhibit the degradation of Mg alloys, among which surface coating is one of the most widely used and effective methods [[21], [22], [23]].

Bone features hierarchical structures from nano to macro scale that enrich with capillaries, cells, nerves, and minerals. Bone regeneration, in which osteoclasts absorb bone and osteoblasts generate new bone, largely depends on its hierarchical structures responding to physiological and mechanical stimulation [24,25]. Hence, the multiscale design of scaffolds is crucial for the temporary substitution of defected bone at the early phase and the speedy restoration of natural bone eventually with biodegradation. Firstly, the macro configuration of the scaffold should fit the anatomical shape of the bone defect to provide smooth alignment and reliable support. Secondly, the scaffold should be composed of interconnected pores to transport nutrients and provide space for bone growth and integration. The stiffness and strength of the scaffold decrease with increasing porosity; thus, Mg scaffolds can accurately mimic the mechanical response of the cortical or cancellus bone by modulating their porosities. In addition to porosity, geometrical features, such as the shape, sizes, and distribution of micropores, significantly influence the mechanical response and bone-repairing effect of scaffolds [26,27]. For instance, porous scaffolds with optimized gradient pores possessed 20 % higher strength than the uniformly distributed ones with similar porosity and stiffness [28]. Additionally, it has been widely recognized that pores in size of 400–600 μm, with negative curvature and smooth transition, are beneficial to bone growth [29].

Laser powder bed fusion (L-PBF) can additively and accurately manufacture Mg scaffolds with customized structures, thus providing the feasibility of precise treatment according to patient-specific needs [30,31]. However, Mg alloys fabricated by L-PBF exhibited a rapid degradation rate due to abundant precipitates in their microstructure. In addition, the interior pores not only expand the surface area but also make scaffolds difficult to obtain reliable coating quality [32,33]. These issues are particularly crucial for medical scaffolds with customized structures and stringent requirements [34]. Previous studies show that high-temperature oxidation (HTO, 525 °C for 8 h) can form a uniform passivated layer of rare earth oxides in several micrometers at the surface of WE43 alloy which significantly decelerates the degradation and simultaneously improves the surface performance, ductility, and biocompatibility [35]. However, animal tests indicate that the passivation effect by HTO may be not sufficient for scaffolds [36]. Layered double hydroxides (LDHs) can create a chemical conversion film of excellent biocompatibility on Mg alloys [[37], [38], [39]]. The LDH film can act as a nanotrap that inhibits corrosion by releasing interlayer anions and capturing corrosion-inducing anions like chlorides through ion exchange [40,41]. Additionally, the LDH film creates rough, hydrophilic nanoscale topographies on the scaffold surface, which provides more functional sites for cell adhesion and differentiation [42].

**Fig. 1:**
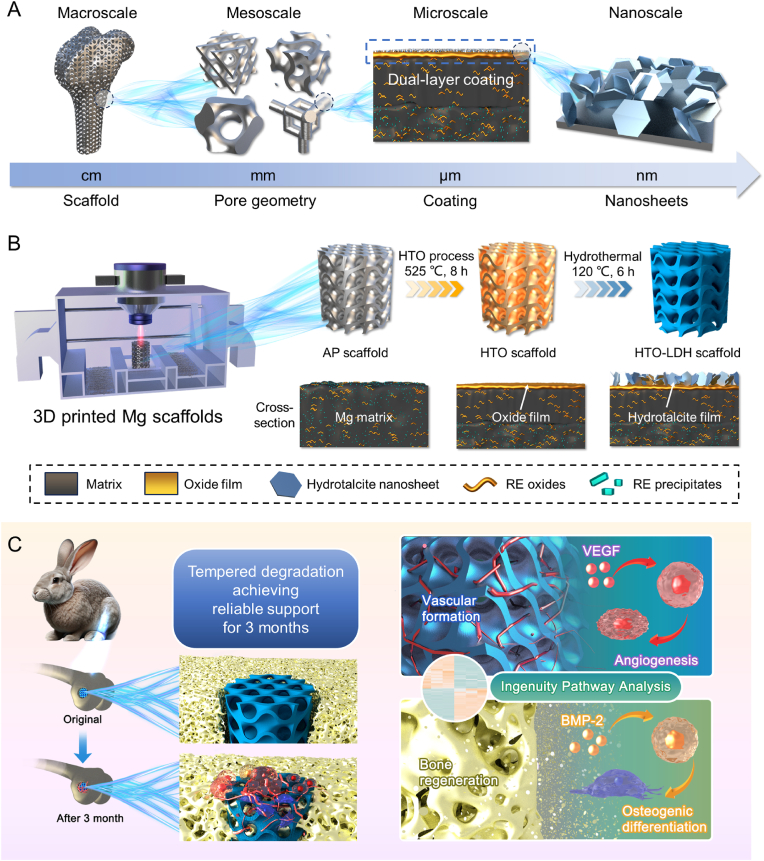
Multiscale Mg scaffold with hierarchical structures (macro-, meso-, and nano-scales) and tempered degradation (microscale) for bone regeneration (**A**) Multiscale design of Mg scaffold from macro to nano (**B**) Synthesis process of multiscale Mg scaffold (**C**) Multiscale Mg scaffold provides reliable support and promotes bone regeneration.

In this work, we present a 3D-printed multiscale functionalized Mg scaffold of WE43 alloy with hierarchical structures (macro-, meso-, and nano-scales) and tempered degradation (microscale) (Fig. 1). This scaffold integrates considerations from macro to nanoscale, from 3D printing precision to Mg ion release kinetics. At the macroscale, the scaffold features anatomic geometries for precise integration with bone defects, achieved through high-precision L-PBF fabrication. The mesoscale pores are devised with optimized curvature and geometries, providing adequate mechanical support as well as promoting cellular proliferation and vascularization. Microscale enhancements comprise a dual-layer coating of HTO and LDH films, creating a protective barrier against rapid degradation at the early phase. The nanoscale textured surface is enhanced with a layered double hydroxide membrane to boost bioactivity and osteointegration. Comprehensive tests both in vitro and in vivo are conducted to characterize the scaffolds and prove the effect from different scales and perspectives. Our methodology holds significant potential for advancing the development of next-generation, multiscale functional biomaterial scaffolds, tailored for effective and customized bone repair.

## 2 Results

### 2.1 Mesoscale design and 3D printing optimized geometrical features and load capacity

**Fig. 2:**
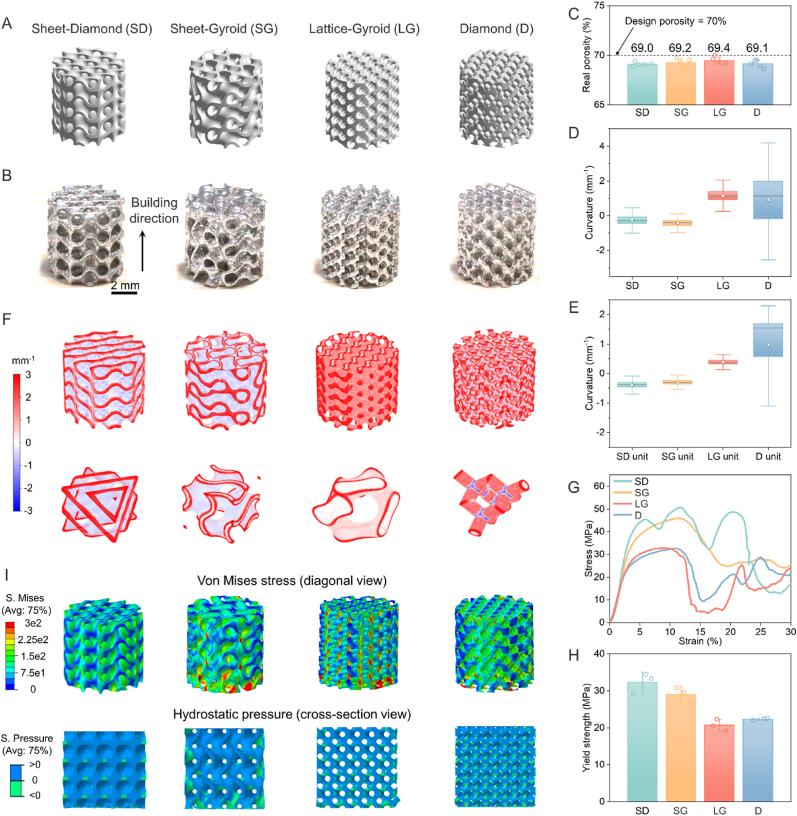
Comprehensive structural analysis of Mg scaffolds for bone implants at macroscale and mesoscale. (**A**) Three-dimensional models of three TPMS porous structures and Diamond porous structure. (**B**) Images of the 3D-printed Mg scaffolds, with a 2 mm scale bar for reference. (**C**) The measured porosities of the scaffolds, with data represented as mean values ± standard deviation (*n* = 5). (**D**, **E**) Mean curvature distribution box plots of internal configurations of the scaffolds (**D**) and unit cells with a side length of 3 mm (**E**). The edges of the structures are excluded in the box plots of mean curvature distribution. Box plots indicate mean (white dot), median (middle line), 25th, 75th percentile (box), and 1.5 × interquartile range (whiskers). (**F**) Mean curvature distributions of the scaffolds and unit cells. The color scale represents the mean curvature values. (**G**) Stress-strain curves of the scaffolds during the compression tests. (**H**) Compressive yield strengths of the scaffolds with various structures. (**I**) Von Mises stress and hydrostatic pressure distributions from the finite element simulations of the scaffolds under a 500 N uniaxial compressive load. The color scale represents the stress levels and volume strain.

Here, we explored three widely recognized triple periodic minimal surface (TPMS) porous scaffolds (Sheet-Diamond, Sheet-Gyroid, Lattice-Gyroid) alongside a popular strut-based porous scaffold (Diamond) for comparative analysis of the influence of pore design on geometrical features and load capacity. Fig. 2A shows the macroscale 3D models of the four scaffolds that boast a cylindrical shape with a diameter and height both 6 mm. The four scaffolds were designed to have the same surface area of 500 mm^2^ and the same porosity of 70 %. Fig. 2B shows pictures of the scaffolds after ultrasonic vibration and chemical etching. More details about fusion quality and surface morphologies of the scaffolds can be found in Supplementary Information.

Fig. 2D–E shows the box plots of the mean curvatures of the four scaffolds and their corresponding unit cells, respectively (additional curvature distribution data can be found in Fig. S3). The SD and SG scaffolds exhibited a more homogenized mean curvature distribution compared to the LG and D counterparts. Moreover, their average and median values skewed towards the more negative end of the spectrum. According to the visual distribution of the mean curvature in Fig. 2F, the internal structures of the SD and SG scaffolds were characterized by a favorable geometric attribute of negative mean curvature values. Conversely, the LG scaffold presented non-negative mean curvature values across its surface. The D scaffold, however, manifested a dichotomy in curvature distribution, displaying highly negative mean curvature values at junction nodes and elevated positive values at non-nodal regions.

Compression tests were conducted on the scaffolds along the building direction (Fig. 2G). Remarkably, the SD scaffold exhibited a compressive strength of 48.8 ± 6.0 MPa despite sustaining a high porosity of 70 %. This strength was approximately 50 % superior to those of the LG and D scaffolds. Fig. 2H illustrates that the SD scaffold boasted the highest compressive yield strength of 32.4 ± 2.7 MPa compared to 22.3 ± 0.3 MPa and 20.7 ± 1.6 MPa of the D and LG scaffolds, meaning that the SD scaffold can endure more stress before mechanical failure. Moreover, the elastic modulus of the SD scaffold was 1502 ± 125 MPa, which matched that of the cancellous bone and is suitable for interior bone filling. More mechanical properties of used scaffolds can be found in Table S1. The Von Mises stress distributions within the finite element models (Fig. 2I) revealed that the LG and D scaffolds were susceptible to severe stress concentration at the junction nodes. By contrast, the SD scaffold exhibited a more uniform stress distribution and lower stress level, suggesting a better load-bearing capacity. According to the calculated distribution of pressure, the SD and SG scaffolds predominantly exhibited positive pressure values on the nodes, indicative of a stretching-dominated deformation mode. Inversely, the LG and D scaffolds displayed markedly negative pressure values, signifying a bending-dominated mode.

In addition to comparing mechanical properties, we further seeded HUVECs on four different scaffolds to investigate the biological effects arising from structural differences. Through SEM imaging and cytoskeleton staining, we observed that the SD and SG groups, with their negative curvature designs, exhibited significantly more endothelial cell adhesion, which is beneficial for subsequent angiogenesis (Fig. S4). To explore the early effects of these four structures on promoting angiogenesis in vivo, we implanted them and performed Microfil angiography after 6 weeks. The results clearly showed that the SD and SG groups, with their negative curvature designs, had significantly more blood vessel formation, consistent with our in vitro findings (Fig. S5). Further immunofluorescence staining for CD31, an endothelial cell marker, corroborated these results (Fig. S6). To delve deeper, we conducted protein assays and found a significant upregulation of genes promoting endothelial cell function on the scaffolds with negative curvature (Fig. S7). This suggests that the negative curvature design of these scaffolds plays a crucial role in enhancing endothelial cell adhesion and promoting angiogenesis.

In summary, L-PBF reliably fabricated Mg scaffolds with customized macroscale shapes and mesoscale pores, demonstrating the flexibility to delicately modulate structure to improve bone repairing performance. The SD scaffold outperformed the other three counterparts in mechanical strength at the equivalent porosity level. It also featured a negative curvature surface, a pore size of approximately 600 μm, and interconnected pores of a 70 % high porosity that all were recommended to facilitate bone regeneration. Moreover, biological evaluations revealed that the SD scaffold significantly enhanced endothelial cell adhesion and subsequent angiogenesis, both in vitro and in vivo. Given these combined attributes of superior mechanical performance and biological activity, ensuing characterizations and analyses were centered exclusively on the SD scaffold for further multi-scale and perspective analyses.

### 2.2 Heat treatment and hydrothermal process regulated micro and nanoscale topography

**Fig. 3:**
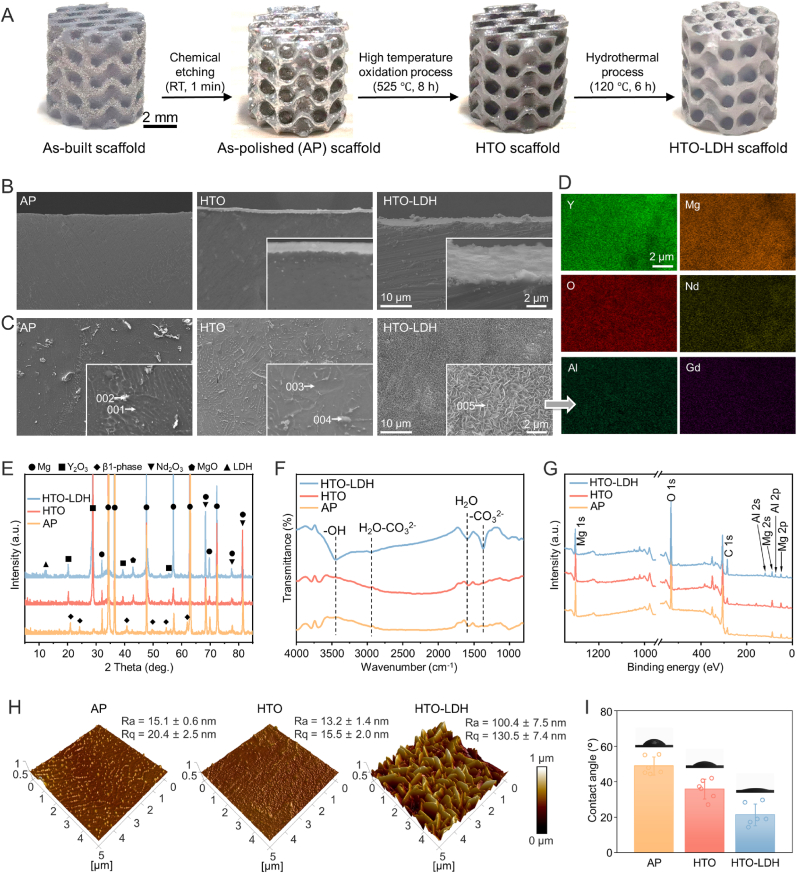
Characterization of Mg scaffolds after different processes. (**A**) The preparation process of the HTO-LDH scaffold. (**B**–**C**) SEM images of the cross-section view and surface morphology. (**D**) Surface EDS mapping of HTO-LDH scaffold. (***E***–**G**) XRD patterns, FT-IR, and XPS spectra of AP, HTO, and HTO-LDH scaffolds. (**H**) AFM images of AP, HTO, and HTO-LDH scaffolds. (**I**) Contact angles of AP, HTO, and HTO-LDH scaffolds. Data are presented as mean values ± s.d. (*n* = 6).

As shown in Fig. 3A, the as-built Mg scaffold of SD design was polished by chemical etching, then endured high-temperature oxidation (HTO) treatment at 525 °C for 8 h [35], and underwent a hydrothermal process at 120 °C for 6 h to further grow Mg-Al layered double hydroxide (LDH) nanosheets. Moreover, we explore the impact of varying hydrothermal process durations (3, 6, 12, and 24 h) on the growth of Mg-Al LDHs on the HTO scaffold surface (More details can be found in “Materials and Methods" and Fig. S2). Fig. S2 characterized the detailed scaffold morphology at various scales after different processes. Furthermore, due to the reactive nature of the Mg alloy, applying hydrothermal treatment to create the LDH membrane coating directly on the as-polished or as-built scaffold without an initial passivation step would lead to corrosion, as demonstrated in Fig. S8. This would compromise the structural integrity of the scaffold and cause rapid degradation in body fluid. Thus, the high-temperature oxidized film is essential as a preparatory layer for the LDH coating. Fig. 3B shows the cross sections adjacent to the surface after different processes. HTO scaffolds showed an oxide film approximately 1.5 μm thick. According to Fig. 3C, a plethora of precipitates exist in the grains and at the grain boundaries at the surface of the AP scaffold. After HTO, oxides majorly composed of Y_2_O_3_ and Nd_2_O_3_ covered the surface, evidenced by the composition measured by EDS (Fig. S9, and Table S2) and the phase tested by XRD in Fig. 3E. Fig. S10 exhibited the cross-section of the scaffold after HTO, in which a continuous oxide film outside and a transition layer between the oxide film and the matrix were observed. Significantly decreased precipitates were observed in the transition layer. More details on the microstructure characterization of AP and HTO scaffolds can be referred to in our previous work [35].

HTO-LDH scaffolds exhibited a composite dual-layer coating on the surface, approximately 3.5 μm thick, consisting of oxide and LDH films. Beyond that, a flake-like vertical LDH nanosheet network, with a lateral size of 600–800 nm, was observed at the surface of the LDH film. EDS elemental mapping verified the growth of Mg-Al LDHs on the surface of the HTO-LDH scaffold (Fig. 3D). Homogeneous distribution of the Al element was observed in addition to Mg, Y, O, and Nd elements on the surface of the HTO-LDH scaffold. The XRD diffraction peak at approximately 12° signified the (003) crystal face of the LDH phase (Fig. 3E).

The characteristic bands in Fourier transform infrared (FT-IR) spectroscopy approximately at 3445 cm^−1^ (stretching vibration of hydroxyl groups), 2945 cm−1 (hydrogen bonding between water and CO_3_^2−^), 1610 cm−1 (stretching vibration of H-O-H), and 1367 cm^−1^ (asymmetric stretching vibration of C=O in CO_3_^2−^) further indicated the incorporation of LDHs on HTO-LDH scaffolds (Fig. 3F). Compared with AP and HTO scaffolds according to Fig. 3G, peaks originating from Al are found in HTO-LDH scaffolds by X-ray photoelectron spectroscopy (XPS), consistent with the EDS results in Fig. 3D. The high-resolution Mg 2p and Al 2p XPS spectra (Fig. S11) revealed the presence of Mg^2+^ (binding energy (BE) at 49.9 eV) and Al^3+^ species (BE at 74.0 eV), confirming the existence of Mg-Al LDH on HTO-LDH scaffolds.

According to atomic force microscopy via the tapping mode (Fig. 3H), HTO-LDH scaffolds demonstrated the highest surface roughness with Ra reaching 100.4 ± 7.5 nm, while no significant difference was observed between the AP and HTO scaffolds. Compared with AP scaffolds, the contact angle decreased at the surface of HTO scaffolds due to the formation of oxides. HTO-LDH scaffolds showed the smallest contact angle of 21.3 ± 6.2° (Fig. 3I), indicating the improved hydrophilicity after the growth of LDH nanosheets. Overall, HTO resulted in a continuous layer of RE oxides, while LDH was successfully deposited on the oxide layer. The microscale dual-layer coating was expected to protect the scaffold from direct contact with corrosive physiological environments, meanwhile, the nano-textured surface promised preferable cell attachment and proliferation.

### 2.3 Tempered biodegradation benefiting from the dual-layer micron coating

**Fig. 4:**
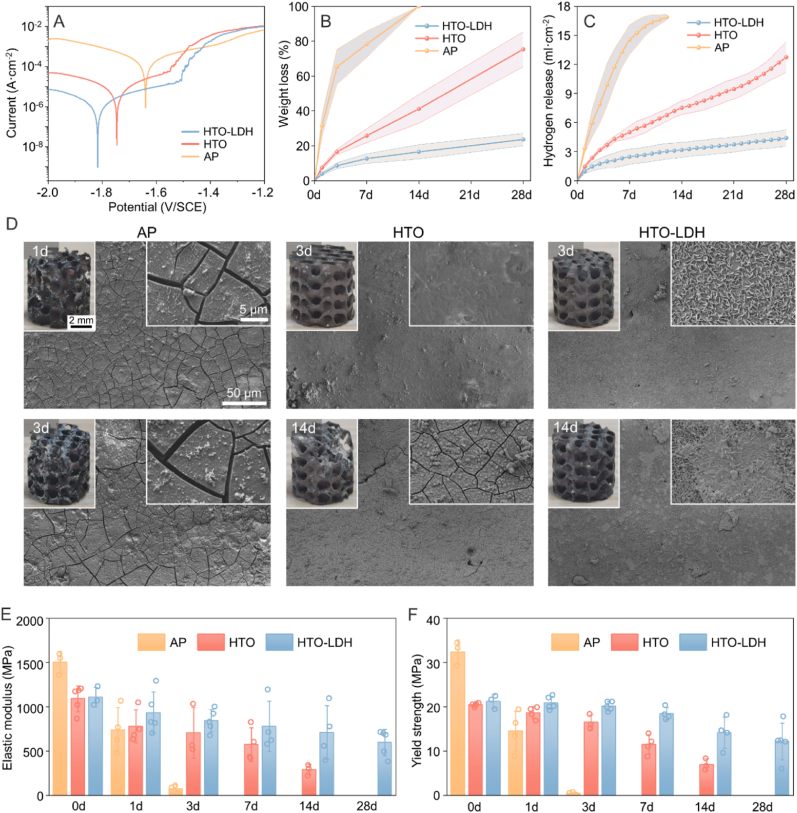
*In Vitro* biodegradation behavior of HTO-LDH scaffolds. (**A**) Potentiodynamic polarization (PDP) curves. (**B**–**C**) Weight loss ratio and released hydrogen volume of AP, HTO, and HTO-LDH scaffolds after 0-, 1-, 3-, 7-, 14-, and 28-day immersion. Data are presented as mean values ± s.d. (*n* = 3). (**D**) Surface morphologies and photos of the AP, HTO, and HTO-LDH scaffolds after 1, 3, and 14 days of immersion. (***E***–**F**) Compressive elastic modulus and yield strength of AP, HTO, and HTO-LDH scaffolds after 0-, 1-, 3-, 7-, 14-, and 28-day immersion in r-SBF solution. Data are presented as mean values ± s.d. (*n* = 3∼5).

In accordance with potentiodynamic polarization curves recorded in revised simulated body fluid (r-SBF) solution, the corrosion current densities (*i*_*corr*_) of the AP, HTO, and HTO-LDH samples were 2.51 × 10^−4^, 9.55 × 10^−6^, and 1.57 × 10^−6^ A cm^2^, respectively, with corresponding corrosion potentials (*E*_*corr*_) as −1.65, −1.72, and −1.82 V/SCE (Fig. 4A). Significant enhancement in corrosion resistance was observed following the HTO treatment and was further augmented with the LDH layer. Additional insights from the electrochemical impedance spectroscopy (EIS) are shown in Fig. S12. Notably, the HTO-LDH samples exhibited an increased impedance modulus |Z| in the Bode plot and an expanded impedance loop at low frequency in the Nyquist plot, indicative of a superior passivation effect.

In addition to the electrochemical test of instantaneous corrosion resistance, the weight loss and the released volume of hydrogen gas were used as indicators of long-term degradation in immersion tests with r-SBF solution at 37 °C for 28 days (Fig. 4B and C). The AP scaffold lost 31.5 ± 6.1 % weight just after 1-day immersion, and completely degraded after 14 days. The weight loss was 16.5 ± 1.5 % and 75.3 ± 10.0 % after 3 and 28 days respectively for the HTO scaffold. By contrast, the HTO-LDH scaffold demonstrated a minimum weight loss of about 23.6 ± 3.6 % and hydrogen gas release of 4.4 ± 0.9 mL cm^−2^ after 28-day immersion, conforming to 0.3 mm year^−1^ approximately. Fig. 4D and Fig. S13 further show the picture of scaffolds after various immersion days. The AP scaffold displayed significant deterioration and surface cracking after 1-day immersion. The structural integrity was severely compromised, with extensive surface pitting noted after 3 days. The HTO scaffold had no obvious degradation after 3-day immersion and maintained structural integrity with notable surface cracks until 14 days, attributed to the protective oxide film. Benefiting from the dual-layer coating, the surface of the HTO-LDH scaffold kept unbroken with rare cracking even after 14 days. The nanosheets on the surface were almost intact after 3 days but were extensively covered with degradation products after 14 days. After 28-day immersion, the structural integrity of the HTO-LDH scaffold was maintained substantially, indicating the robust shield against degradation; by contrast, the HTO scaffold collapsed and lost 75.3 ± 10.0 % weight, suggesting the deteriorated protection in challenging conditions. Moreover, as shown in Fig. S14, the quantity of Al^3+^ and Mg^2+^ ions released from the HTO-LDH scaffolds are also characterized by an inductively coupled plasma optical emission spectrometer (ICP-OES). The results show that the cumulative release of Al ions is 6.2 ± 0.6 μg/L after 1 day and 128.8 ± 30.5 μg/L after 28 days of immersion in r-SBF solution. These levels are well within the biologically permissible range [43]. Additionally, as shown in Fig. S15, the Al content in the Mg scaffolds is 0.054 ± 0.004 wt%, equals to 54 ± 4 μg per scaffold, further confirming its negligible risk.

The damage to the structure by degradation considerably comprised the load-bearing capacity of the scaffold as shown in Fig. 4E–F. A significant decline was observed in the AP scaffold's elastic modulus and yield strength, by 95 % and 98 %, respectively, after 3-day immersion. The HTO scaffold experienced a 73 % and 66 % reduction after 14 days, while the HTO-LDH scaffold recorded a decline of 46 % and 43 % after 28 days. In general, the dual-layer micron coating by HTO and LDH remarkably protected the Mg scaffold from rapid degradation and contributed to reliable mechanical support for a longer time during implantation.

### 2.4 Enhanced cell adhesion: interplay of tempered degradation and nanotopography

**Fig. 5:**
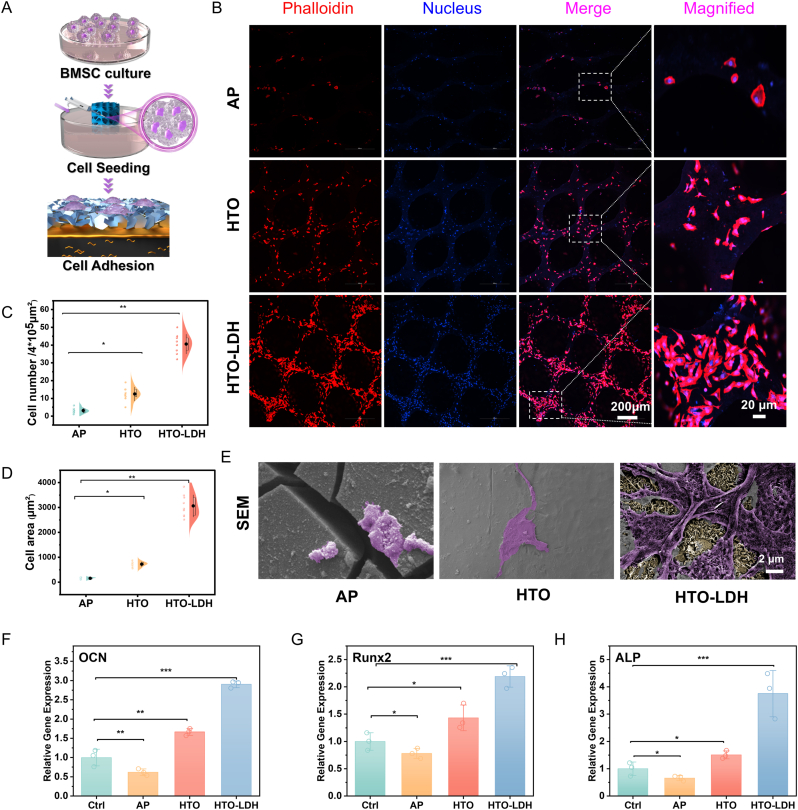
*In Vitro* cell adhesion of HTO-LDH scaffolds. (**A**)Schematic diagram, (**B**) CLSM observations, **(C**) the corresponding quantitative assay of cell number, and (**D**) the cell area of BMSCs cultured on the scaffolds for 12 h (actin filament is stained red, while the cell nuclei are stained blue). (**E**) SEM observations. (**F-H**) Osteogenic gene (OCN, ALP, and Runx2) expression of hBMSCs cultured with CTRL, AP, HTO, and HTO-LDH scaffolds evaluated by qPCR. Data are presented as mean values ± s.d. (*n* = 3). ∗p < 0.05, ∗∗p < 0.01, ∗∗∗p < 0.001 (one-way ANOVA).

Upon initial observations made 12 h post-cell seeding, we examined the morphology of the BMSCs cultured on the scaffold to assess the state of cell adhesion (Fig. 5A). The HTO-LDH scaffold manifested the most substantial cell adhesion, conspicuously higher than the other groups (Fig. 5B). In contrast, the AP scaffold exhibited the least cell adhesion. A more granular observation revealed a pronounced dispersion of cell morphology in the HTO-LDH scaffold. Quantitatively, the HTO-LDH scaffold had a cell count that was 3.26-fold greater than the HTO scaffold and a striking 13.04 times that of the AP scaffold (Fig. 5C). Moreover, BMSCs cultured on the HTO-LDH scaffold presented an elongated form, with extended filopodia anchored firmly onto the scaffold's surface. The cell area of the HTO-LDH scaffold was 4.25 and 19.89 times those of the HTO and AP scaffolds respectively (Fig. 5D). Further morphological insights were garnered using Scanning Electron Microscopy, providing pivotal details about cell-material interactions (Fig. 5E). BMSCs growing on the HTO-LDH scaffold displayed a flattened polygonal shape, punctuated by extended lamellipodia anchored onto hydrotalcite nanosheets.

Additionally, we investigated the influence of the scaffold on the key osteogenic gene expression in bone marrow-derived mesenchymal stem cells via quantitative reverse transcription-polymerase chain reaction (Fig. 5F–H). Compared to the control group (Ctrl), the HTO-LDH group cells by one-day post-cultivation manifested markedly upregulated genes, including Alkaline Phosphatase (ALP), Osteocalcin (OCN), and Runt-related Transcription Factor 2 (Runx2). Conversely, the AP group demonstrated a slightly declining trend relative to the Ctrl group, with ALP, Runx2, and OCN expression dropping by 34.2 %, 21.9 %, and 41.4 % respectively. The expression levels in the HTO group were 2.87-fold for both OCN and ALP and 1.83-fold for Runx2 when juxtaposed with the AP group. Hence, combined with cell adhesion data, our results suggest that the rapid degradation of AP scaffolds resulted in excessive Mg ions and increased pH, thus adversely affecting cell adhesion and osteogenic differentiation. With the superior surface condition and tempered degradation, the HTO-LDH group had an OCN expression that was 4.18 times, ALP that was 5.7 times, and Runx2 that was 2.81 times that of the AP group, manifesting exceptional cell adhesion and osteogenic differentiation potential.

### 2.5 *In vitro* osteogenesis facilitated by tempered degradation

**Fig. 6:**
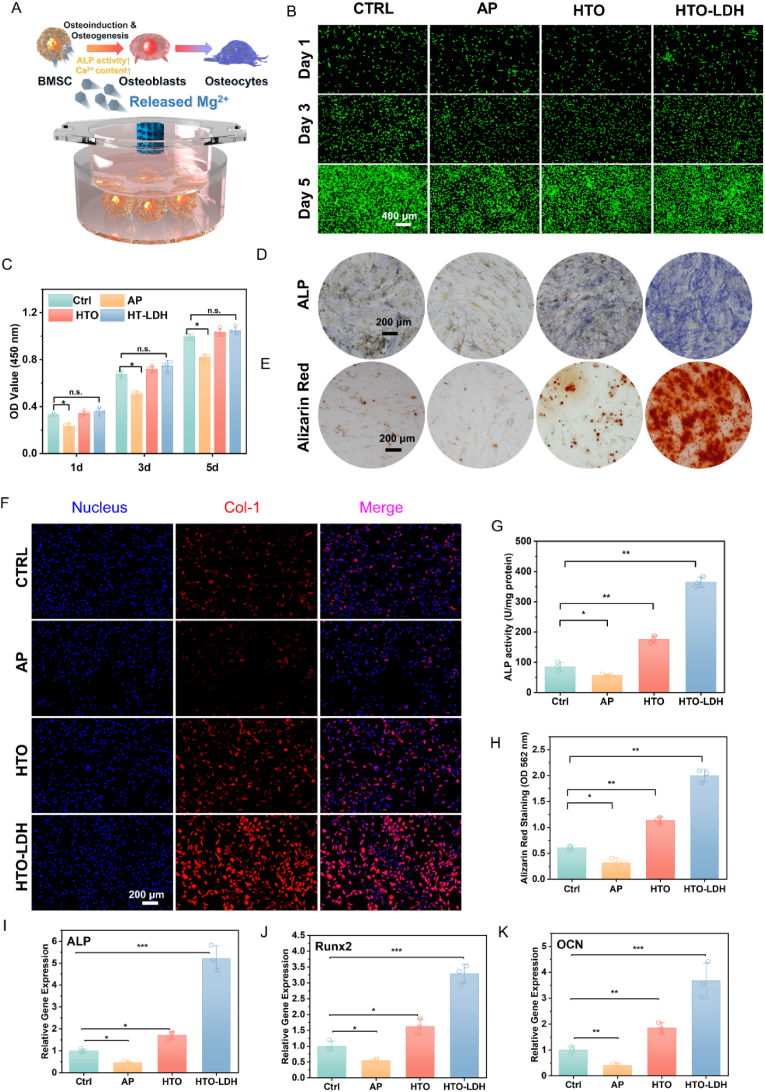
*In vitro* biocompatibility and osteogenesis of HTO-LDH scaffolds. (**A**) Schematic diagram, (**B**) Calcein-AM/PI (live/dead) staining, Scale bar: 400 μm. **(C**) CCK-8 assay. (**D**) Alkaline phosphatase and (**E**) Alizarin red S staining of hBMSCs cultured with CTRL, AP, HTO, and HTO-LDH scaffolds. Scale bar: 200 μm. (**F**) Immunostaining of Col-1 after 14 days' osteogenic differentiation of hBMSCs cultured with CTRL, AP, HTO, and HTO-LDH scaffolds (bar = 200 μm). (**G-H**) Quantitative analysis results of Alkaline phosphatase and Alizarin red S staining. (**I-K**) Osteogenic gene (OCN, ALP, and Runx2) expression of hBMSCs cultured with CTRL, AP, HTO, and HTO-LDH scaffolds evaluated by qPCR. Data are presented as mean values ± s.d. (n = 3). ∗p < 0.05, ∗∗p < 0.01, ∗∗∗p < 0.001 (one-way ANOVA).

Transwell chambers were employed to cultivate cells in a scaffolded environment (Fig. 6A). Initially, we qualitatively and quantitatively evaluated the biocompatibility of the Mg scaffolds using human bone marrow mesenchymal stem cells (hBMSCs). The calcein-AM/propidium iodide (PI) double staining experiments were conducted, facilitating direct in vitro visualization of biocompatibility on 1, 3, and 5 days (Fig. 6B). For hBMSCs cultured within the scaffolds at identical time points, the AP group exhibited relatively fewer live cells. Almost no dead cells were discernible across all groups. However, there was negligible variation in the quantity of live cells in the AP group when comparing the HTO, HTO-LDH, and Ctrl groups. Furthermore, according to the CCK-8 assay results, no detrimental impact on cell viability was observed for the HTO and HTO-LDH groups compared to the Ctrl group on days 1, 3, and 5 (Fig. 6C). In contrast, the AP group exhibited decreased cell viability relative to the Ctrl group. It suggests that the rapid degradation of the AP scaffold results in high Mg ion concentration, which is not conducive to hBMSC proliferation, though it does not induce significant cell death.

The osteogenic differentiation potential of the scaffolds was further evaluated by monitoring alkaline phosphatase (ALP) activity and the formation of a mineralized matrix. Relative to the AP and HTO scaffolds, the HTO-LDH group manifested an intensively robust ALP staining after 14 days of cultivation, underscoring that the HTO-LDH coating augmented osteogenic differentiation in hBMSCs. The HTO group displayed a modest osteogenic effect, whereas the osteogenic effect of the AP group seemed somewhat inhibited. Quantitative data for ALP activity aligned with the staining results. Notably, the ALP activity in the HTO-LDH group was 2.08 times that of the HTO group and 6.4 times that of the AP group (Fig. 6D and G). After 21 days of cultivation, Alizarin Red S staining was employed to evaluate osteogenic differentiation. This analysis unveiled an abundance of mineralized nodules in the HTO-LDH group, which contrasted sharply with the scant mineralization observed in the Ctrl and AP scaffold groups. Quantitative analysis of the dissolved Alizarin Red S indicated a significant 47.18 % reduction in mineralized staining for the AP group when compared to the Ctrl group. Both the HTO-LDH and HTO groups exhibited values markedly higher than the Ctrl group, with the HTO-LDH group reaching 6.25 times the AP group, underlining its osteogenic advantage (Fig. 6E and H).

Nevertheless, we conducted immunofluorescence studies and quantitative polymerase chain reaction (qPCR). Immunofluorescence staining corroborated that culturing hBMSCs in the presence of HTO-LDH scaffolds enhanced bone formation. After 14 days, cells cultured in the HTO-LDH environment exhibited a pronounced upregulation in the expression of the bone-healing-related protein - collagen (Col 1), demonstrated by the most intense positive fluorescent staining (Fig. 6F). Consequently, on day 14, qPCR was employed to quantitatively gauge the expression levels of ALP, OCN, and Runx2 in hBMSCs. The expression levels of ALP, OCN, and Runx2 in the AP group were marginally reduced compared to the Ctrl group (Fig. 6I–K). This observation, consistent with ALP staining and Alizarin Red staining results, suggests that rapid degradation of AP might curtail the expression of osteogenesis-related genes. The HTO-LDH group manifested the highest expression levels for the three indicators, markedly surpassing all three groups. It is inferred that fast degradation and excessive degradation products inhibit the expression of these genes. Cumulatively, the observed mRNA and protein expression levels underscore that HTO-LDH coatings significantly enhance the osteogenic differentiation of hBMSCs. These outcomes not only attest to the Mg scaffolds' inherent bioactivity but also highlight the tempered degradation by the microscale dual-layer coating in creating a conducive milieu for bone regeneration.

### 2.6 Promoting angiogenesis through tempered degradation

**Fig. 7:**
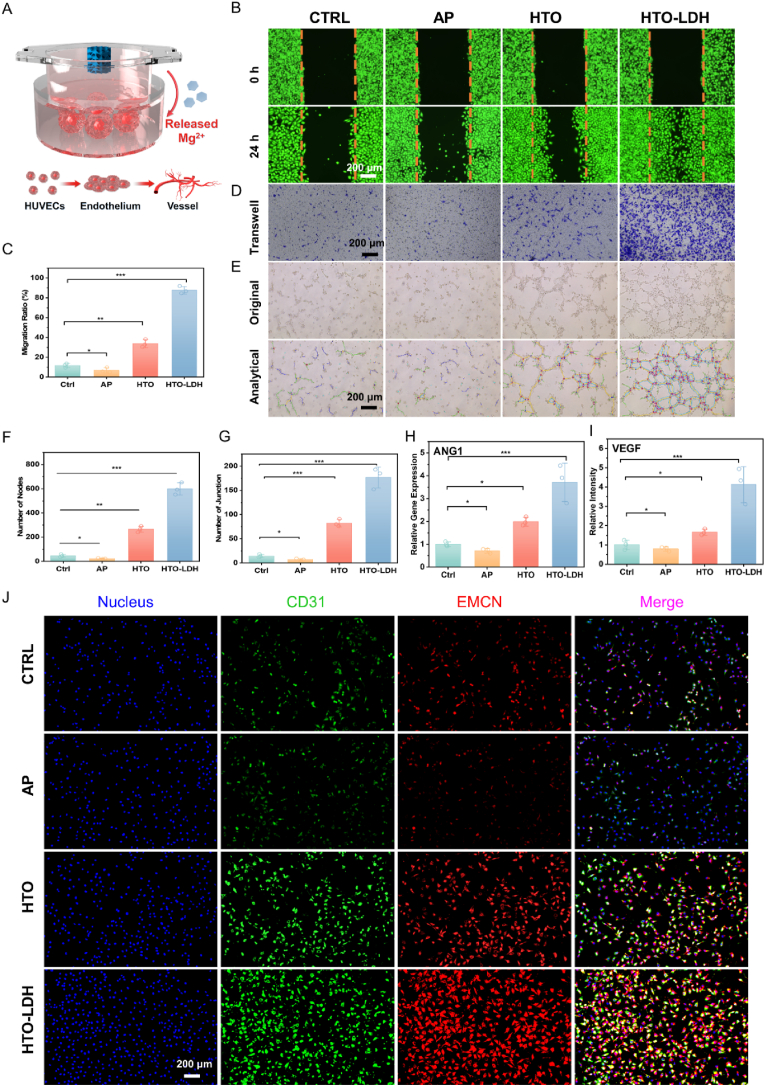
*In Vitro* Angiogenesis of HTO-LDH Mg Alloy Scaffolds. (**A**) Schematic diagram, (**B-C**) Optical microscope images and further quantitative analysis results (ImageJ 1.52q1.52 vsoftware) of scratch assay evaluating the migration activity of HUVECs cultured with CTRL, AP, HTO, and HTO-LDH scaffolds. (**D**) Migrated HUVECs cultured with CTRL, AP, HTO, and HTO-LDH scaffolds stained with crystal violet in Transwell assay. (**E**) Matrigel experiment results accessing the vessel generation capability of HUVECs cultured with CTRL, AP, HTO, and HTO-LDH scaffolds. Scale bar: 200 μm. (**F-G**) Quantitative analysis of the Matrigel experiment results counting the number of joint and total length formed by HUVECs using ImageJ 1.52q1.52v software. (**H-I**) Angiogenic gene (AGN1 and VEGF) expression of HUVECs cultured with CTRL, AP, HTO, and HTO-LDH scaffolds evaluated by qPCR. Data are presented as mean values ± s.d. (n = 3). ∗p < 0.05, ∗∗p < 0.01, ∗∗∗p < 0.001 (one-way ANOVA). (**J**) Immunostaining of CD31 and EMCN of HUVECs cultured with CTRL, AP, HTO, and HTO-LDH scaffolds (bar = 200 μm).

To assess the angiogenic potential of scaffolds in vitro, we utilized human umbilical vein endothelial cells (HUVECs) to perform wound healing assays, Transwell migration assays, and tube formation assays (Fig. 7A). Post a 24-h incubation, a significant narrowing of the wound gap was observed in the HTO-LDH treated group. The increased wound closure area indicates an augmented cell migration rate, suggesting that the incorporation of LDH significantly bolstered cellular migration. Quantitative analysis further corroborated this, with the HTO-LDH group exhibiting a 13.03-fold higher migration rate of HUVECs than the AP group, and a 2.59-fold increase compared to the HTO group (Fig. 7B–C). The Transwell assays, utilizing crystal violet staining to visualize migrated cells, reflected the same trend. As depicted in Fig. 7D, the HTO-LDH group displayed a significantly higher number of migrating cells as well, further reinforcing the role of HTO-LDH in fostering cell migration to set the stage for angiogenesis. The direct assessment of angiogenic levels with the matrix gel experiment (tube formation assay) revealed that the HTO-LDH group formed distinctly more capillary-like structures on the matrix gel, presenting the highest number of tube structures (Fig. 7E). Subsequent quantification illustrated a substantial increase in the number of nodes and junctions in the HTO-LDH group, 25.24 and 26.42 times that of the AP group, and 2.15 and 2.25 times that of the HTO group, respectively (Fig. 7F–G).

To delve deeper into the angiogenic mechanism of HTO-LDH, we subjected the treated HUVECs to qRT-PCR and immunofluorescence assays. The AP scaffold exhibited a decrease in VEGF and ANG1 gene expression levels compared to the Ctrl group (Fig. 7H–I). However, the VEGF and ANG1 gene expression in the HTO group were superior to that of the Ctrl group. The HTO-LDH group showcased the highest levels of VEGF and ANG1 gene expression, which were 4.12 and 3.71 times that of the Ctrl group, respectively. On the foundation laid by HTO, the HTO-LDH further moderated the rapid degradation of the Mg scaffold, and the judicious release of Mg ions due to tempered degradation played a significant role in promoting angiogenesis. H-type vessels, characterized by the expression of CD31 and Endomucin (EMCN), have been implicated in the bone formation process. The HTO-LDH group exhibited markedly brighter fluorescence for CD31 and EMCN (Fig. 7J). Overall, it is confirmed that HTO-LDH demonstrates outstanding angiogenic promotion capability, majorly explained by the adequate physiological stimulus from tempered degradation.

### 2.7 Elucidating osteogenic and angiogenic pathways: insights from ingenuity pathway analysis

**Fig. 8:**
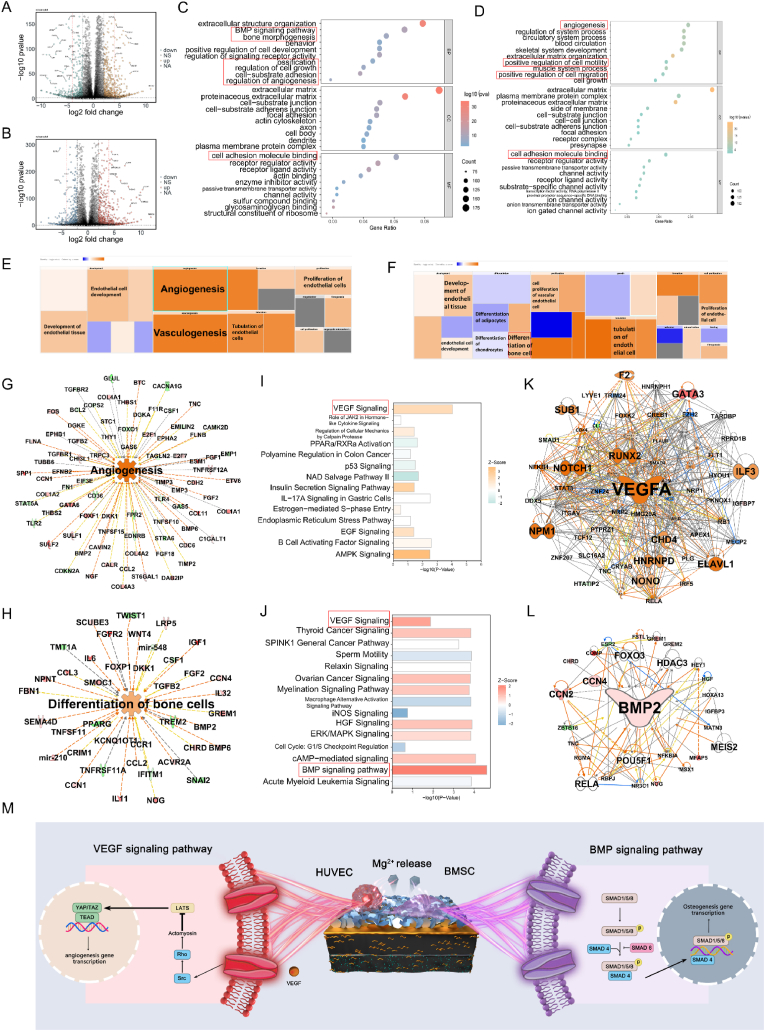
Potential mechanisms underlying the osteogenic and angiogenic characteristics. The volcano plot of the differentially expressed genes identified by transcriptome sequencing of (**A**) HUVECs and (**B**) hBMSCs, the up-regulated genes are marked in red/orange color, and the down-regulated genes are marked in green/blue color. Cutoff: P value < 0.05 and |log2 FC| > 1. GO analysis of differentially expressed genes of (**C**) hBMSCs and (**D**) HUVECs. Relationships between the activation and inhibition of disease and function are determined by the expression profiles of differentially expressed of (**E**) HUVECs and (**F**) hBMSC. Orange indicates activation of a disease or functional state (z-score >0), blue indicates inhibition of a disease or functional state (z-score <0), and gray indicates uncertainty regarding a disease or functional state (z-score could not be calculated). (**G-H**) Activation and inhibition relationships between transcription factors and functions (angiogenesis and differentiation of bone cells). Red indicates upregulated gene expression, green indicates downregulated gene expression, and the color gradation indicates the intensity of the effect. The dotted lines indicate indirect interactions. Canonical signal pathways identified by IPA® of (**I**) HUVECs and (**J**) hBMSC. PPI network of genes mapping the correlation of (**K**) VEGF and angiogenesis, (**L**) BMP2 and differentiation of bone cells. (**M**) Schematic diagram.

As illustrated in Fig. 8A–B, differential gene expression between HTO-LDH scaffold-treated and untreated hBMSCs/HUVECs was observed by transcriptome sequencing analysis, with active genes marked in red or orange, and silent genes in blue and green in the heatmap. Volcano plot analysis revealed 3734 differentially expressed genes in hBMSCs and 4214 in HUVECs (p < 0.05 and |log2 fold change| > 1), including 1901 upregulated and 1833 downregulated genes in hBMSCs, and 2224 upregulated and 1990 downregulated genes in HUVECs (Fig. 8A–B). Gene set enrichment analysis, utilizing multiple databases including Gene Ontology (GO) and the Kyoto Encyclopedia of Genes and Genomes (KEGG), identified pathways involved in the cellular response to the scaffolds. In hBMSCs, GO enrichment analysis suggested the involvement of the BMP signaling pathway, bone morphogenesis, ossification, cell-substrate adhesion, regulation of angiogenesis, and cell adhesion (Fig. 8C). KEGG analysis highlighted several osteogenesis-related pathways, such as the Wnt signaling pathway (Fig. 8C). For HUVECs, GO analysis indicated involvement in angiogenesis, positive regulation of cell migration, cell growth, and cell adhesion molecule binding (Fig. 8D), with the KEGG analysis suggesting involvement in several angiogenesis-related pathways, such as the VEGF signaling pathway (Fig. 8D).

Further exploration of key molecular mechanisms was conducted through Ingenuity Pathway Analysis. The functional module results highlighted several osteogenic and angiogenic-related functional modules that were activated, particularly those associated with the differentiation of bone cells and angiogenesis (Fig. 8*E*–F). Predictive molecular interaction networks generated by IPA shed light on pivotal genes that might mediate the effects of the scaffold. Genes such as IGF1, FGFR2, and IL-6 were notably overexpressed, potentially driving the differentiation of bone cells (Fig. 8H). Simultaneously, genes like FOXF1 and SPP1 were significantly upregulated, likely facilitating angiogenesis (Fig. 8G). Canonical pathway analysis using IPA pointed to the BMP signaling pathway and VEGF signaling pathway as two critical activated pathways (Fig. 8I–J). Differential genes were plotted on pathway maps as shown in Figs. S19 and S20. The interaction network centered on the core molecules BMP2 and VEGFA from the differential genes further clarified the contribution of activated BMP and VEGF signaling pathways to the enhanced osteogenesis and angiogenesis afforded by the HTO-LDH scaffold (Fig. 8K–L). Based on the initial IPA analysis results, we conducted additional assessments of key proteins in the VEGF and BMP pathways. These assessments were performed both in vitro and in vivo to validate the activation of these critical pathways. The protein assay results confirmed significant activation of the VEGF and BMP pathways (Figs. S21 and S22), consistent with the earlier IPA analysis findings (Figs. S19 and S20). This supports the conclusion that the HTO-LDH scaffold promotes osteogenesis and angiogenesis through the activation of the VEGF and 10.13039/100018052BMP pathways. These results illuminate that the activation of these signaling pathways potentially contributes to the HTO-LDH scaffold's enhanced capacity for bone formation and vascular development, which can be attributed to hierarchical structures enhancing cell bioactivity (in macro-, meso-, and nano-scales) and tempered degradation releasing Mg ion adequately (in microscale).

### 2.8 *In vivo* bone regeneration: synergy of hierarchical structures and tailored degradation

**Fig. 9:**
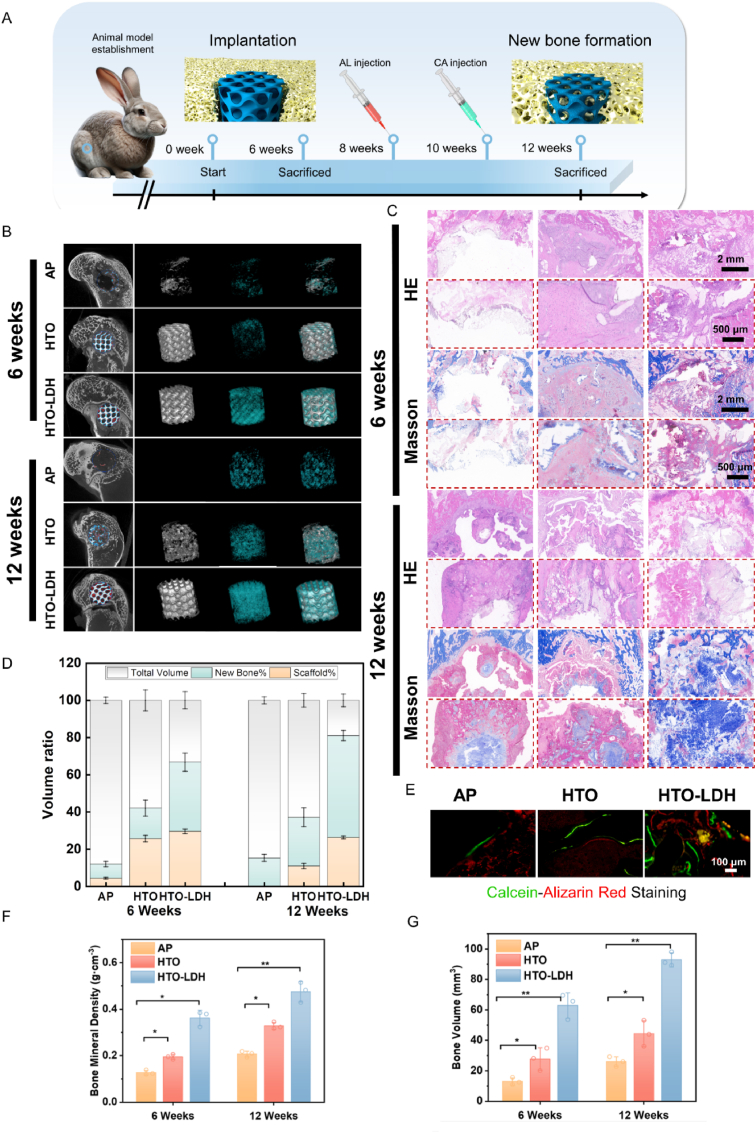
*In vivo* bone regeneration of HTO-LDH scaffolds. (**A**) Schematic diagram of animal experiments. (**B**) The coronal and 3D reconstructed Micro-computed tomography (Micro-CT) scanning images of skull defects and regenerated bone tissue induced by AP, HTO, and HTO-LDH scaffolds. (**C**) H&E and Masson trichrome staining of the regenerated bone induced by AP, HTO, and HTO-LDH scaffolds were shown at low and high magnification. (**D**)Analysis of the remaining amount of scaffold and the proportion of new bone volume. (**E**) Representative fluorescence imaging. (**F-G**) Quantitative morphometric analysis of the bone volume and bone mineral density of the newly formed bone using micro-CT analysis. ∗p < 0.05, ∗∗p < 0.01, ∗∗∗p < 0.001, (N = 3, one-way ANOVA).

Mg scaffolds were implanted into cylindrical defects in rabbit femoral condyles. The timeline and arrangement for the in vivo experiments are depicted in Fig. 9A. Firstly, throughout the 12-week study period, all rabbits maintained good health without observable adverse effects in vital organs such as the heart, liver, spleen, lungs, and pancreas, as indicated in Figure S16. According to micro-computed Tomography (micro-CT) scans and corresponding analyses (Fig. 9B), the AP group lost its original structure after 6 weeks, with some bone regeneration observed. For the HTO group, at 6 weeks post-implantation, the scaffold surface became slightly rough with minor degradation, but the overall structure was largely unaffected; small amounts of bone tissue were found within the pores, tightly adhering to the pore walls. Meanwhile, the HTO-LDH scaffold remained intact with more bone formation compared to the other groups. The quantity of new bone formed increased progressively with time post-implantation. After 12 weeks, the AP group had almost completely degraded, with increased new bone formation observed compared to the 6-week mark. However, for the HTO group, after 12 weeks, the original scaffold structure was lost, but some scaffold pieces remained along with a certain amount of bone regeneration (Fig. 9B). In the HTO-LDH group, the best anti-degradation effect and bone regeneration capacity were still observed after 12 weeks post-implantation.

Quantitative analysis of the micro-CT results showed that the bone volume in the HTO-LDH group was 3.57 times that of the AP group, and the bone mineral density was 2.3 times greater (Fig. 9F and G). Furthermore, by the 12-week post-implantation mark, the HTO-LDH scaffold had only degraded by 11.13 %, preserving its structural integrity, while the HTO group experienced 63 % degradation, losing its complete structure (Fig. 9D). The AP group had completely degraded after 12 weeks. These findings indicate that the HTO-LDH group successfully balances anti-degradation capability with the promotion of bone regeneration, making it a highly promising candidate for bone repair materials. Histological staining, including Hematoxylin and Eosin (H&E) and Masson's trichrome, demonstrated the growth of new bone and fibrous tissue within the pores of all three types of scaffolds (Fig. 9C), corroborating the micro-CT findings by indicating the formation of new bone trabeculae. Using sequential fluorescent labeling, with injections of calcein-AM and alizarin red at two-week intervals, revealed that the mineral apposition rate was the greatest in the HTO-LDH group (Fig. 9E). At 6 and 12 weeks, the HTO-LDH group both generated more new bone compared to the AP and HTO groups, corroborating the superior bone regenerative effect at the tissue level.

## 3 Discussion

This study introduces a customized Mg scaffold with hierarchical structures and tempered degradation, designed to address the biological, physical, and chemical demands of high-performance bone regeneration at varying scales.

At the macroscale, the outline of the Mg scaffold is tailored to conform to the specific anatomical characteristics of the bone defect. It is fabricated with optimized laser powder bed fusion parameters using WE43 powder, ensuring reliable fusion and accurate formation. Such high-quality and precision scaffolds provide essential structural support and promote integration with the surrounding bone tissue. It also aids in efficiently distributing mechanical loads, mimicking the mechanical functionality of natural bone.

The mesoscale design of the scaffold emphasizes load capacity and internal geometric features, including strength, modulus, curvature, pore size, and interconnectivity. Utilizing smoothly connected pore units generated by a Sheet-Diamond-based TPMS structure, the scaffold achieves a compressive strength of approximately 49 MPa while maintaining a high porosity of 70 %, which is indicative of its ability to ensure mechanical support during the healing process. The internal pores of the scaffold, characterized by negative mean curvature, facilitate cell migration and adhesion. An optimal pore size of around 600 μm, along with high interconnectivity, is critical for nutrient transportation, waste removal, and vascularization. These features also provide space for cell proliferation and extracellular matrix deposition, thereby actively interacting with the bone's microenvironment and promoting effective tissue ingrowth and vascularization. To further validate the advantages of the negative curvature design, we conducted comparative experiments with scaffolds featuring different structural designs. Our results demonstrated that scaffolds with negative curvature significantly improved neovascularization compared to those with suboptimal designs. This was evidenced by enhanced endothelial cell adhesion and migration, as well as increased vascular formation in vivo, as confirmed by CD31 immunofluorescence staining and Microfil angiography. The negative curvature design of the scaffold thus plays a crucial role in promoting early-stage vascularization, which is essential for successful bone regeneration.

At the microscale, the Mg scaffold's design prioritizes tempered degradation, ensuring a proper release rate of Mg ions, structural integrity, and mechanical support during the bone healing process. A microscale dual-layer coating, comprising an oxide film and a hydrotalcite film, is applied to the scaffold's surface through a combination of heat treatment and hydrothermal processing. This dual-layer coating provides effective protection against rapid degradation of Mg. In comparative tests, the HTO-LDH scaffold with this coating exhibited a weight loss of approximately 24 % after 28 days immersed in r-SBF solution at 37 °C. In contrast, the uncoated AP scaffold degraded completely within about 10 days, while the HTO scaffold, coated only with the oxide film, fully degraded after roughly 28 days. The comparison by in vitro test results also demonstrated that the HTO-LDH scaffold exhibited significantly enhanced angiogenic and osteogenic properties, evidenced by a 6.4-fold increase in ALP activity, a 6.25-fold increase in Alizarin Red S staining, and a 13.03-fold increase in cell migration ratio. The superior performance of the HTO-LDH scaffold in promoting vascular and bone tissue regeneration can be attributed to its controlled degradation and the resulting change in the microenvironment. 10.13039/100014337Furthermore, the microscale dual-layer coating ensured tempered degradation of the scaffold in vivo, maintaining its structural integrity and mechanical support. 12 weeks post-implantation, the HTO-LDH scaffold had degraded only by 11.13 %, whereas the HTO scaffold, coated only with the oxide film, underwent 63 % degradation, losing its major structure. In contrast, the uncoated AP scaffold completely degraded within 12 weeks.

The nanoscale textured surface of the Mg scaffold is enhanced with layered double hydroxide nanosheets. These nanosheets display tens of nanometers in thickness and 400–800 nm in length approximately, achieved by optimizing the hydrothermal processing condition. The HTO-LDH scaffold with these nanosheets demonstrates superior hydrophilicity with a contact angle of about 20°. Furthermore, the nanosheets contribute to a rough nanoscale topography with a surface roughness Ra roughly 100.4 nm, thereby increasing the number of adsorption sites available for cell adhesion and osteogenic differentiation.

RNA sequencing results revealed the synergistic effects at the nano and micro scales, promoting cell adhesion and activating the BMP and VEGF pathways, which are crucial for vascular regeneration and osteogenic differentiation, thereby facilitating bone repair. Building upon the macro- and meso-scales, which cater to the anatomic shape and customized pores of Mg scaffolds, the microscale and nanoscale designs of the scaffold guarantee a hierarchical structure and tempered degradation. In vivo comparison conducted over 12 weeks in rabbits confirmed the outstanding potential of this multiscale Mg scaffold as a bone repair scaffold. Moreover, we conducted additional protein assays based on the initial IPA analysis results. These assays were performed both in vitro and in vivo to validate the activation of key pathways. The protein assays confirmed significant activation of the VEGF and 10.13039/100018052BMP pathways, supporting our initial findings that the HTO-LDH scaffold promotes osteogenesis and angiogenesis through these pathways. These results are presented in the supporting information, enriching our understanding of the in vivo cellular mechanisms underpinning bone regeneration.

The proposed HTO-LDH scaffold, through its synergistic multiscale functionality, significantly enhanced bone volume and mineral density, promoting new bone formation while maintaining structural integrity and mechanical support.

Our findings indicate that the effectiveness of the layered double hydroxide (LDH) coating is not isolated but interdependent with the structural and functional attributes at different scales. The integration of these multiple elements is crucial for the scaffold's overall performance. The macroscopic design tailored for anatomical fit, the mesoscale architecture promoting mechanical support and vascularization, the microscale dual-layer coating ensuring controlled degradation, and the nanoscale enhancements facilitating cell adhesion and differentiation, all collectively contribute to the scaffold's efficacy. The necessity of this complex design is evident as each component is optimized to address specific biomedical needs, ensuring the efficacy and functionality of the final scaffold.

Our research reveals that the Mg scaffold, featuring a hierarchical structure and tempered degradation, offers sufficient mechanical support while promoting cell adhesion, proliferation, and differentiation. This leads to accelerated bone regeneration following implantation in critical-sized femoral condyle bone defects in rabbits. Our approach shows great promise for the development of the next generation of multiscale functional biomaterial scaffolds for effective customized bone repair.

## CRediT authorship contribution statement

**Zehui Lv:** Writing – review & editing, Writing – original draft, Project administration. **Bo Peng:** Writing – review & editing, Writing – original draft, Investigation. **Yu Ye:** Investigation, Methodology. **Haojing Xu:** Investigation, Formal analysis. **Xuejie Cai:** Software, Methodology. **Jinge Liu:** Conceptualization. **Jiabao Dai:** Software, Methodology. **Yixin Bian:** Data curation. **Peng Wen:** Supervision, Funding acquisition. **Xisheng Weng:** Supervision, Funding acquisition.

## Ethics approval and consent to participate

All animal experiments were conducted in strict compliance with the Animal Care and Use Committee guidelines, adhering to the principles outlined in the Guide for the Care and Use of Laboratory Animals by the National Institutes of Health, USA, and following the Peking Union Medical College guidelines for animal welfare. The ethical lot number for this project is: JS-2625.

## Declaration of competing interest

The authors declare the following personal relationships which may be considered as potential competing interests: Yu Ye is currently employed by Hefei Boya Maite Biomaterials Co., Ltd. Peng Wen is an editorial board member for Bioactive Materials and was not involved in the editorial review or the decision to publish this article.

## Materials and Methods

### Mesoscale design and fabrication of Mg scaffolds

Triple periodic minimal surface (TPMS) and related structures are widespread in natural biological systems [44]. TPMS scaffolds provide excellent geometric structures to describe the biological form of the human skeleton. Numerous studies have shown that the curved surfaces of TPMSs contribute to enhanced plasma membrane elongation during cell crawling and spreading [45,46]. Here, we focus on three specific TPMS structures (Sheet-Diamond, Sheet-Gyroid, and Lattice-Gyroid), members of the TMPS family [46]. Three TPMS structures in this study can be described by the implicit equations below:

(1) Sheet−Diamond:−c<cosXcosYcosZ+sinXsinYsinZ+sinXcosYsinZ+cosXsinYsinZ<c

(2) Sheet−Gyroid:−c<sinXcosY+sinYcosZ+sinZcosX<c

(3) Lattice−Gyroid:sinXcosY+sinYcosZ+sinZcosX<c

Where *c* is a constant related to the porosity. *X =* 2*πx/α*, *Y =* 2*πy/α*, *Z =* 2*πz/α*, *α* is a constant related to the unit cell size. The polynomial coefficient is set to be equal in the *x*, *y*, and *z* dimensions to ensure the isotropic shape and mechanical properties of the porous structures. Different *α* and *c* values are selected to ensure the three TPMS structures (cylinder with a diameter of 6 mm and height of 6 mm) have a surface area of 500 mm^2^ and 70 % porosity (±5 % deviation is tolerant). For Sheet-Diamond, Sheet-Gyroid, and Lattice Gyroid, *α* values are 2.8, 2.25, and 1.1, respectively, and *c* values are 0.32, 0.47, and −0.6, respectively. The code to create these structures is available on GitHub (https://github.com/Bop2000/Code4STL_and_Curvature). SolidWorks 2021 software is used to create the Diamond cylinder structure with a diameter of 6 mm, a height of 6 mm, a surface area of 500 mm^2^, and 70 % porosity (±5 % deviation is tolerant).

The performance of the powder has an extensive influence on the formation quality of the 3D-printed products. WE43 alloy powder that comprised 3.50 % Y, 2.28 % Nd, 1.18 % Gd, 0.07 % Zr, 0.20 % Zn, 0.02 % Mn, and balance Mg, spherical powders with few satellite particles are observed, suggesting good flowability (Fig. S1A). The 3D printing of WE43 alloy is executed using a compact laser powder bed fusion (LPBF) machine (BLT S210, China) equipped with a laser having a spot diameter of 70 μm. The optimized processing parameter with laser power of 60 W, scanning speed of 600 mm/s, layer thickness of 20 μm, and hatch space of 70 μm is used in this study. The WE43 powder bed underwent preheating to 200 °C before the initiation of the melting process. A zig-zag exposure pattern is deployed for the inner hatch region, incorporating a 67° rotation (α) in each layer to enhance structural integrity and uniformity. The relative density of the WE43 scaffold is 99.82 ± 0.07 %, as evidenced in Fig. S1C. After the printing process, a chemical etching procedure involving a solution of 5 % nitric acid, 5 % hydrochloric acid, and balance C_2_H_5_OH (applied at room temperature for 1 min) is employed to effectively remove adhered powder particles. As shown in Fig. S2, the polished scaffold structure remains connected without obstruction and has a smooth interlayer transition. Such attributes indicate a scaffold that not only stimulates bone ingrowth but also enlarges exposed functionalized surface areas [47].

### Mechanical tests and finite element simulation analysis

Cylindrical porous structures, each measuring 6 mm in both diameter and height, are employed to assess the mechanical properties of the scaffolds. Mechanical compression tests are executed utilizing an Instron machine equipped with a 10 kN load cell, operating at a crosshead speed of 1 mm min^−1^ and room temperature. Testing is aligned parallel to the scaffolds' building direction, with a minimum of three samples evaluated to affirm the tests' reliability and consistency.

The theoretical compressive mechanical response of the scaffolds is analyzed using the finite element method by Abaqus software. In the simulations of the finite element models, a uniform static load of 500 N is applied to the scaffolds in the axial direction as compressive stress, similar to the axial compressive stress on load-bearing bones in the human body [48,49].

In addition, compression finite element simulations are conducted utilizing a high-performance computing environment equipped with a 32-core, 64-thread Intel Xeon Gold 6226 R Processor and facilitated through ABAQUS software. Cylinder porous structures with a diameter of 6 mm and height of 6 mm are utilized to conduct finite element analysis. The FEA is based on the same rigid-cylinder and deformable-implant-structure model. Material properties are assumed isotropic for the analysis. The elastic modulus of linear elastic materials is set to 40 GPa, Poisson's ratio as 0.3, and density as 1.8 g cm^−3^ using the steady-state static model. On the upper surface of the model, an axial 500 N compressive load is applied to the bottom surface uniformly.

### Curvature estimation

The mean curvature of the structures is estimated with a Python-based implementation using a package trimesh. The code returns the discrete mean curvature measure of a sphere centered at a point as detailed in ‘Restricted Delaunay triangulations and normal cycle'- Cohen-Steiner and Morvan [50]. The radius of the local sphere used for curvature estimation is set as 0.1 mm. This neighborhood size strikes a balance between smooth and localized estimations of curvature. The code is available on GitHub (https://github.com/Bop2000/Code4STL_and_Curvature).

### Preparation of HTO and HTO-LDH scaffolds

Following the 3D printing of Mg scaffolds, an initial chemical etching is employed to eliminate adhered powder particles on the as-built scaffold which led to an expanded surface area and accelerated the degradation rate. This process involves a solution of 5 % nitric acid, 5 % hydrochloric acid, and balance C_2_H_5_OH at room temperature for 1 min, followed by ultrasonic cleaning in pure C_2_H_5_OH, yielding what we term the as-polished scaffold. Subsequently, a high-temperature oxidation treatment (HTO) is executed in circulating air at 525 °C for 8 h, utilizing a digitally controlled electrical furnace with a constant heating rate of 10 °C per minute. Quenching is achieved by immediate immersion in room-temperature water. This procedure results in the formation of a microscale oxide film on the surface of the as-polished scaffold, denoted henceforth as the HTO scaffold (Fig. S3).

The fabrication of the HTO-LDH scaffold is achieved by promoting the growth of Mg-Al LDH nanosheets on HTO scaffolds through a hydrothermal process. The HTO scaffolds are meticulously cleaned with deionized water and absolute ethanol, each involving a 5-min ultrasound bath, before being placed in a Teflon-lined stainless-steel container. For the LDH film preparation, a solution of 50 mL Al(NO_3_)_3_ (0.02 M) added with 600 μL NaOH (10 M) is introduced to the container. The reaction kettle is maintained at 120 °C for 6 h, after which the samples are taken out, ultrasonically cleaned for an additional 5 min, and rinsed with deionized water. These samples are denoted as the HTO-LDH scaffold. We also explore the impact of varying hydrothermal process durations (3, 6, 12, and 24 h) on the growth of Mg-Al LDHs on the HTO scaffold surface. As shown in Fig. S2, the density of Mg-Al LDHs on the scaffold surface augmented in tandem with the extension of hydrothermal reaction time. However, it is noteworthy that pitting corrosion is pronounced after 12 and 24 h of hydrothermal treatment. In contrast, a 3-h treatment yields an insubstantial growth of Mg-Al LDHs. Consequently, a 6-h duration is selected as the optimal hydrothermal process time to balance the growth of Mg-Al LDHs and mitigate scaffold corrosion.

### Materials characterization

The morphology and element compositions of the scaffolds are examined with field-emission scanning electron microscopy (FESEM, Zeiss Gemini-300, Germany) and energy dispersive spectroscopy (EDS) at an accelerating voltage of 15 kV. The phase compositions are identified via X-ray diffraction (XRD, Bruker D8 Advance, USA), utilizing Cu Kα radiation set at 40 kV and 40 mA. Fourier transform infrared spectra are recorded by a NETZSCH X70 spectrometer (FT-IR, NETZSCH, Germany) from 650 to 4000 cm^−1^ with 2 cm^−1^ resolution. X-ray photoelectron spectroscopy (XPS, Thermo ESCALAB 250Xi, USA) is employed to ascertain the surface chemical compositions and the valence states of the elements present on the scaffold surfaces. The surface morphology and roughness are studied with atomic force microscopy (AFM, Bruker Dimension Icon, USA) via the tapping mode. The wettability of the scaffold surfaces is determined by a surface contact angle goniometer (Data Physics OCA 25, Germany). The porosities of the scaffolds are quantified by employing the ethanol displacement method [51]. The scaffold residue in the solution after immersion was melted down using HNO_3_, then the ion concentration was characterized via an inductively coupled plasma optical emission spectrometer (ICP-OES, Agilent 5110, USA).

### Electrochemical measurements and in vitro degradation tests

The instantaneous corrosion behavior of 3D-printed Mg alloys is evaluated using an electrochemical workstation in revised simulated body fluid (r-SBF) at room temperature. A three-electrode system, comprising a specimen as the working electrode, platinum as the counter electrode, and saturated calomel as the reference electrode, is employed. Samples are immersed for 30 min to stabilize the open circuit potential (OCP). Electrochemical impedance spectroscopy (EIS) is performed from 10^5^ Hz to 0.01 Hz with a 10 mV amplitude. Potentiodynamic polarization (PDP) tests are conducted at a 1 mV/s scanning rate between −2 and −1 V. Each test is executed in triplicate. Corrosion current density (*I*_*corr*_) is determined by linear fit and Tafel extrapolation within a ±50 mV range around the corrosion potential (*E*_*corr*_).

*In vitro* degradation tests are conducted inside an incubator with r-SBF solution at 37 °C for 4 weeks. The compressive properties of the degraded Mg scaffolds are determined after degradation tests. The corroded surface morphologies of the Mg alloys are inspected by SEM. The weight loss ratio is calculated after the removal of degradation products using a CrO_3_ solution after 1, 3, 7, 14, and 28 days of immersion. The hydrogen evolution in volume is collected with an inverted alkali burette.

### Scaffold Cell Adhesion Experiment

All samples were subjected to ethylene oxide sterilization before commencing the assays to ensure aseptic conditions. Each scaffold variant was carefully positioned in individual wells of a 24-well plate (Corning, USA) and seeded with a cell density of 100,000 cells per well. Following a 12-h incubation period, cells were fixed using 4 % paraformaldehyde, stained with phalloidin and DAPI as per the instructions of the staining kit, and then visualized using a Confocal Laser Scanning Microscope (LSCM, LSM800, Zeiss, Germany). The subsequent quantitative analysis of cell number and area was performed using ImageJ software. Post-seeding, scaffolds with adhered cells underwent a stepwise dehydration process after fixation in an electron microscopy-grade fixative and were then critical point dried. Scanning Electron Microscopy (SEM) was employed to further elucidate the intricacies of cell-material interactions.

### In Vitro Cytocompatibility Assessment

Subsequent experiments utilized a 24-well Transwell plate with a pore size of 3 μm (Corning, #3415, USA) to co-culture materials and cells, observing the effects of the scaffolds on cellular behavior. After seeding 10,000 cells in the lower chamber for 12 h, scaffolds were introduced to the upper compartment. On days 1, 3, and 5, cell viability was assessed using a CCK-8 kit and live/dead cell staining according to the kit's instructions. The absorbance at 450 nm was measured (Varioskan Flash; Thermo Fisher Scientific, USA) to evaluate cell viability. The live/dead cell staining results were then visualized using the LSCM (LSM800, Zeiss, Germany).

### In Vitro Osteogenic Differentiation

The method employed was consistent with that of the scaffold cell adhesion experiment. After 24 h of cultivation, cells were enzymatically detached, and total RNA was isolated using Trizol (Invitrogen, Carlsbad, CA, USA). cDNA synthesis was conducted using the TaKaRa PrimeScript RT reagent Kit with gDNA Eraser (Takara, Shiga, Japan) following the manufacturer's protocols. Gene expression levels were quantified utilizing the TaKaRa TB Green® Premix Ex Taq™ (Takara, Shiga, Japan), and relative gene expression was calculated via the ΔΔCT method. These assays were performed in triplicate to ensure data reliability.

Following a 14-day co-culture, ALP activity was measured at an absorbance of 405 nm using a microplate reader (Varioskan Flash; Thermo Fisher Scientific, USA). Cells were fixed in 4 % paraformaldehyde stained for ALP activity and observed under a microscope. Additionally, mineralized nodule formation was evaluated using Alizarin Red S (ARS) staining. After fixation, 1 mL of ARS solution was added to each well, incubated for 30 min, washed with PBS, and observed microscopically. The OD values at 450 nm were obtained after extraction with acetic acid and neutralization with ammonium hydroxide.

### Quantitative Real-Time PCR (qRT-PCR) Analysis

Cell culture for RNA extraction was carried out for 14 days, analogously to the in vitro cytocompatibility evaluation. HUVECs and human Bone Marrow Stromal Cells (hBMSCs) were harvested and subjected to RNA isolation. The extracted RNA was reverse transcribed, and gene expression levels were quantified employing TaKaRa TB Green® Premix Ex Taq™ (Takara, Shiga, Japan) in accordance with the manufacturer's instructions. Relative gene expression was computed using the ΔΔCT method, with each sample assayed in triplicate to ensure the robustness of the data. The assays were meticulously conducted as per the provided reagent guidelines, with gene-specific primers detailed in the supplementary materials.

### Scratch Assay

HUVECs were cultured to confluence in the lower chamber of a 6-well Transwell plate with a pore size of 3 μm (Corning, #3492, USA). A scratch was made using a 200 μL pipette tip, and cells were then cultured in a serum-free medium. Scaffolds were placed in the upper chamber and co-cultured for 24 h. Images of the scratch were captured at 0 and 24 h to record wound closure. Cell migration rate was calculated as follows: Migration rate (%) = (A0 - An)/A0 × 100 %, where A0 represents the initial wound area and A is the remaining wound area at the time of measurement.

### Transwell Assay

HUVECs were seeded in the upper chamber of a 24-well Transwell plate with 8  μm pores (Corning, NY, USA), while scaffolds were placed in the lower chamber, and cultured in serum-free medium. After 24 h, non-migratory cells were removed using a disposable medical swab, and migrated cells were fixed and stained with 0.1 % crystal violet. Images of migrated cells were captured at random fields.

### Tube Formation Evaluation

Overnight-thawed Matrigel matrix was homogeneously spread across a 24-well plate and incubated at 37 °C to allow gelation. Human Umbilical Vein Endothelial Cells (HUVECs) at a seeding density of 3 × 10^4 were carefully planted onto the solidified gel. Subsequently, scaffolds of interest were introduced into the upper compartment of a 6-well Transwell plate with a 3 μm pore size (Corning, #3492, USA), to assess their influence on tubular structure formation by the endothelial cells.

### Immunofluorescence Staining

HUVECs and hBMSCs were seeded at a concentration of 2 × 10^5 cells/mL in 6-well plates. On day 7 post-cultivation, the cells were rinsed with PBS, fixed with 4 % paraformaldehyde, permeabilized with 0.1 v/v % Triton X-100, and blocked with 10 % bovine serum albumin. Overnight incubation at 4 °C with primary antibodies was followed by thorough washing with TBST and subsequent incubation with IgG secondary antibodies (Abcam). Post-TBST washes, cells were examined under a fluorescence microscope. Key antibodies included those targeting COL-I (Abcam), CD31 (Abcam), and EMCN (Abcam).

### Transcriptome Sequencing

Mirroring the qRT-PCR protocol, cells were directly seeded onto the scaffolds and cultured for 14 days before the total RNA was extracted. Eukaryotic mRNA was enriched using magnetic beads with Oligo (dT), then fragmented using interruption agents. The fragmented mRNA served as templates in the subsequent synthesis of single-strand cDNA using six-base random primers. A double-strand cDNA synthesis was then executed to form the double-stranded cDNA library. This library was purified, repaired, and PCR-amplified after selecting the fragment sizes. Post-validation using an Agilent 2100 bioanalyzer, the constructed library was sequenced using an Illumina sequencer. Moreover, network and pathway analyses were performed with Ingenuity Pathway Analysis software (IPA®, v01-04, QIAGEN Redwood City, CA, USA), providing a comprehensive insight into the genetic interactions and pathways engaged.

### Multiplex Assay Using Luminex Technology

Cell and tissue samples from the femoral condyle surrounding the implanted scaffolds were harvested. Samples were immediately snap-frozen in liquid nitrogen and stored at −80 °C until further analysis. For protein extraction, the tissue samples were homogenized in RIPA buffer (Thermo Fisher Scientific, Waltham, MA, USA) containing protease and phosphatase inhibitors (Sigma-Aldrich, St. Louis, MO, USA). The homogenates were centrifuged at 14,000 rpm for 15 min at 4 °C, and the supernatants were collected for protein quantification. To quantify the levels of multiple key proteins involved in angiogenesis and osteogenesis, we employed Luminex xMAP technology using a multiplex assay kit (Bio-Rad, Hercules, CA, USA).

### Femoral Condyle Bone Defect Animal Model and Scaffold Implantation

A femoral condyle bone defect model was established using New Zealand White rabbits. Under anesthesia, the skin was sterilized, and the distal femur was accessed through a blunt dissection, exposing the femoral condyle. Utilizing a slow-rotating bur equipped with physiological saline, a bone tunnel was gradually expanded to a diameter of 6 mm and a depth of 6 mm. Each group's scaffolds were then meticulously implanted into the defect sites. Wounds were sutured with precision to ensure postoperative recovery. All animal experiments were conducted in strict compliance with the Animal Care and Use Committee guidelines, adhering to the principles outlined in the Guide for the Care and Use of Laboratory Animals by the National Institutes of Health, USA, and following the Peking Union Medical College guidelines for animal welfare.

### Microfil Perfusion

After 6 weeks of implantation, the rabbits were euthanized with an overdose of sodium pentobarbital. The thoracic cavity was opened, and the descending aorta was cannulated. The vascular system was flushed with heparinized saline (100 U/mL) to remove blood clots. Subsequently, a radiopaque silicone rubber compound (Microfil MV-122, Flow Tech Inc., Carver, MA, USA) was prepared according to the manufacturer's instructions and injected through the cannula. The compound was allowed to polymerize at room temperature for 24 h.

### Micro-Computed Tomography (micro-CT) Scanning and Analysis

At 6 and 12 weeks post-implantation, femoral condyle specimens from all experimental animals were harvested and fixed in 4 % paraformaldehyde for 48 h. Specimens were scanned using micro-computed tomography (micro-CT; Siemens, Germany). Three-dimensional reconstruction was performed using Mimics software (Materialise, Leuven, Belgium), allowing the quantification of the new bone volume fraction (BV/TV) and bone mineral density (BMD).

### Histological Assessment

To track new bone formation, fluorescent labeling of mineralized bone tissue was employed using sequential administration of calcein-AM (10 mg/kg, Sigma-Aldrich) and alizarin red (30 mg/kg, Sigma-Aldrich) injected intramuscularly into the rabbits' femurs, with a two-week interval between injections. Post two weeks from the last injection, animals were euthanized, and femurs were collected and fixed in 10 % formalin for three days. Specimens were then embedded for hard tissue sectioning and examined under a fluorescence microscope (Leica, DMi8, Germany) to capture fluorescent images. Histological evaluation of the sections was conducted after HE staining and Masson's trichrome staining. Bone tissues were fixed using 4 % paraformaldehyde, decalcified with 5 % ethylenediaminetetraacetic acid, and embedded in paraffin for pathological sectioning. Hematoxylin and eosin (H&E) staining characterized the bone defects, and imaging was performed under an optical microscope. Masson's trichrome stain was applied to assess the maturity of collagen within the samples.

#### Statistical Analysis

Data are expressed as mean ± standard deviation (S.D). Statistical comparisons were made by one-way ANOVA (for multiple comparisons): ∗p < 0.05, ∗∗p < 0.01, ∗∗∗p < 0.001.
