# Supplementary material for: Bolstered bone regeneration by multiscale customized magnesium scaffolds with hierarchical structures and tempered degradation
Source: Bioact Mater. 2025 Jan 3;46:457–75. doi: 10.1016/j.bioactmat.2024.12.002 (PMC11755084; doi:10.1016/j.bioactmat.2024.12.002)
Supplement: Multimedia component 1 [file mmc1.docx]

Supplementary Information for

**Bolstered bone regeneration by** **multiscale customized magnesium scaffolds with** **hierarchical structures and** **tempered degradation**

Zehui Lv^#^, Bo Peng^#^, Yu Ye, Haojing Xu, Xuejie Cai, Jinge Liu, Jiabao Dai, Yixin Bian,

Peng Wen*, Xisheng Weng*

**Supplementary Notes:**

The Mg scaffolds were additively manufactured by L-PBF using WE43 alloy powders. Only tiny blowholes were observed at the cross-section of the scaffold struts and the relative density at the cross-section was 99.82 ± 0.07%, suggesting good fusion quality and reliable performance (Figure S1). Figure 2B shows pictures of the scaffolds after ultrasonic vibration and chemical etching that were conducted to remove the adhered powder particles on the surface. The actual porosities of the scaffolds were quantified through a liquid displacement technique, revealing a consistency with the designed value (Figure 2C). Figure S2A demonstrated the surface morphologies of the scaffolds at various magnification factors. The internal pores of the scaffolds remained interconnected without obstruction and had a smooth interlayer transition.

**Supplementary Figures:**


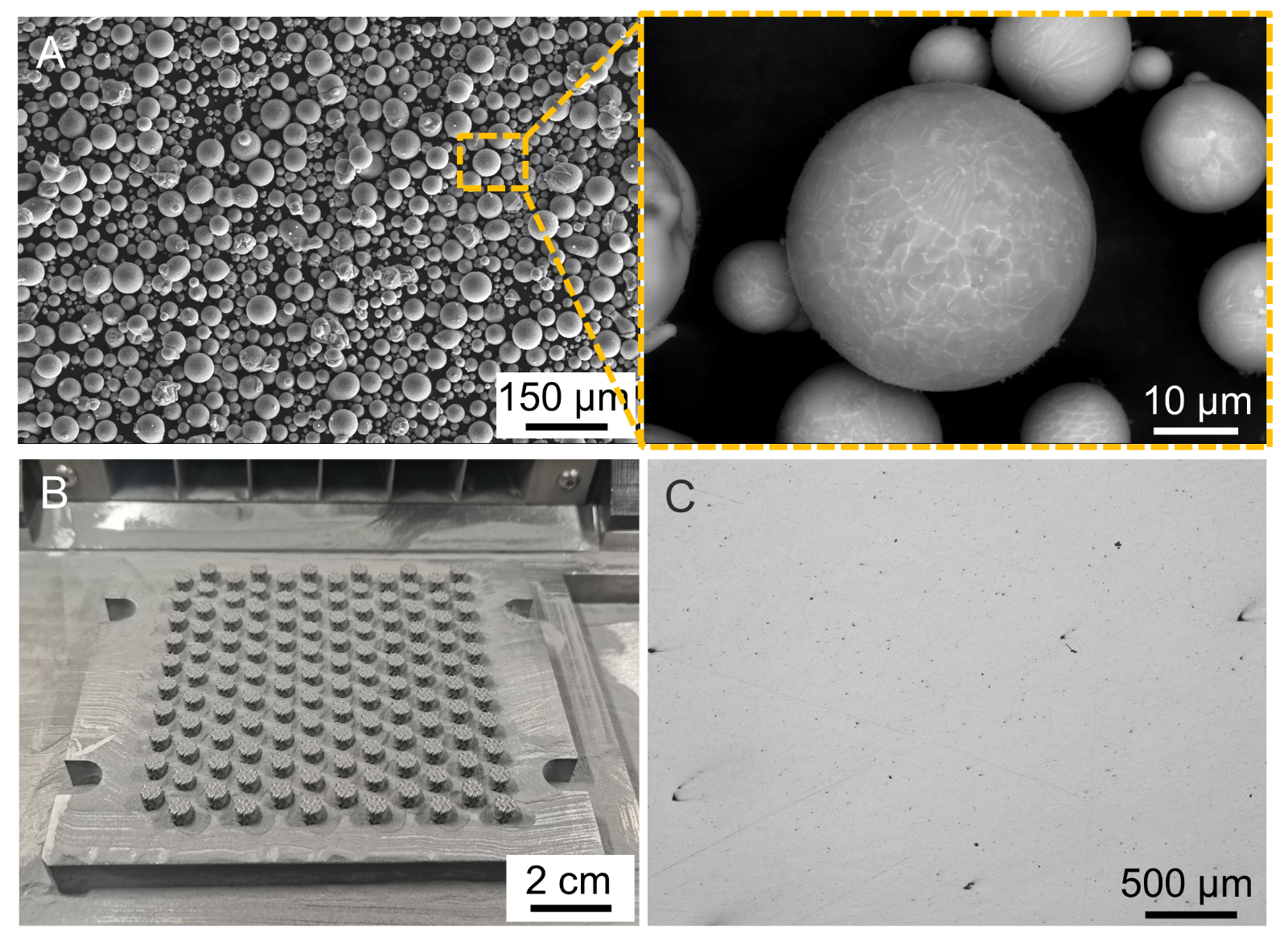


1. (**A**) Scanning electron microscopy (SEM) images of the WE43 alloy powders used in the laser powder bed fusion (LPBF) process. (**B**) Image of 3D-printed Mg scaffolds by laser powder bed fusion. (**C**) Optical image of the cross-section of the 3D-printed Mg alloy.


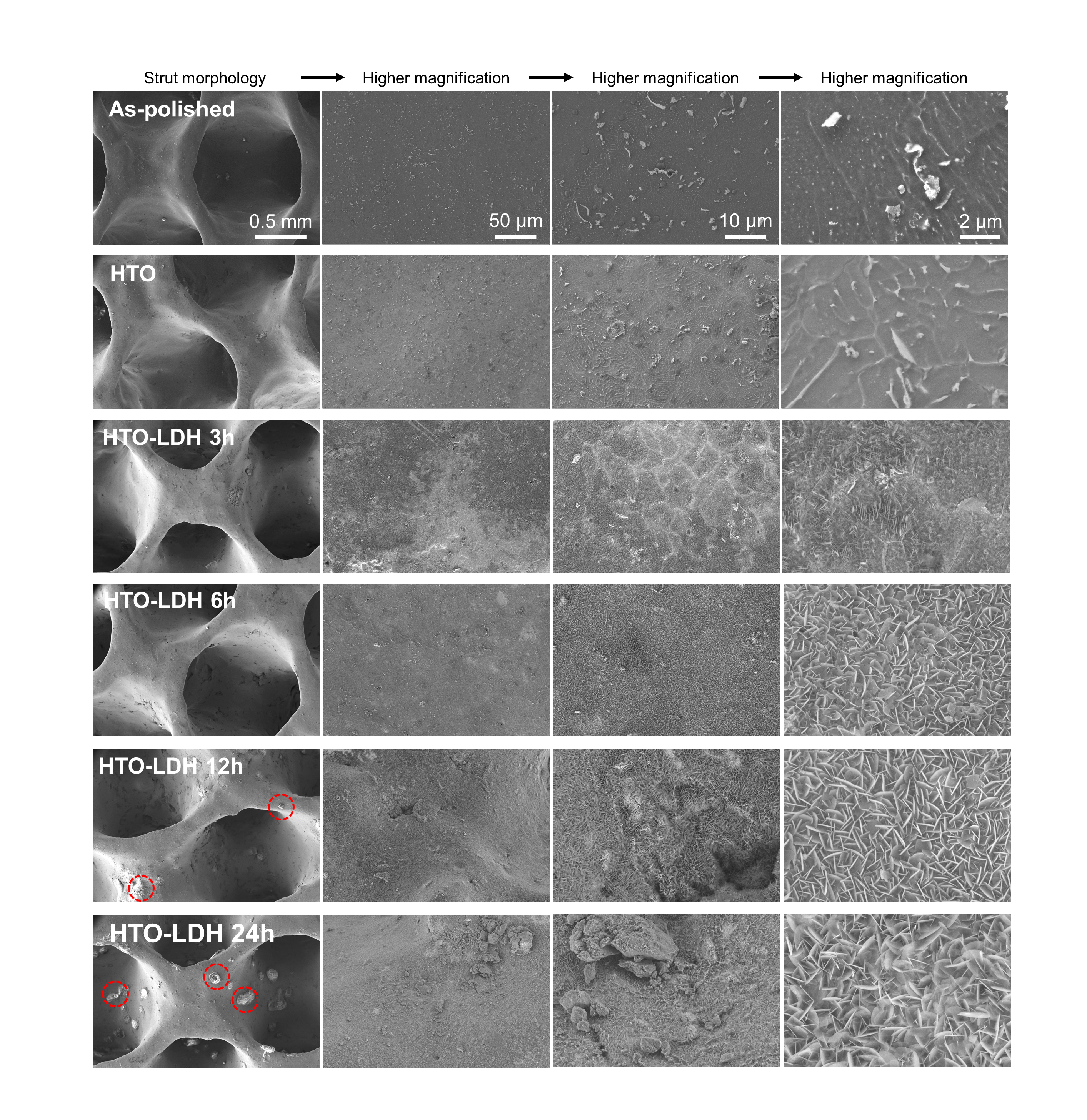


1. Surface morphologies of AP, HTO, and HTO-LDH (Hydrothermal process time: 3h, 6h, 12h, 24h) scaffolds under different magnifications observed by SEM.


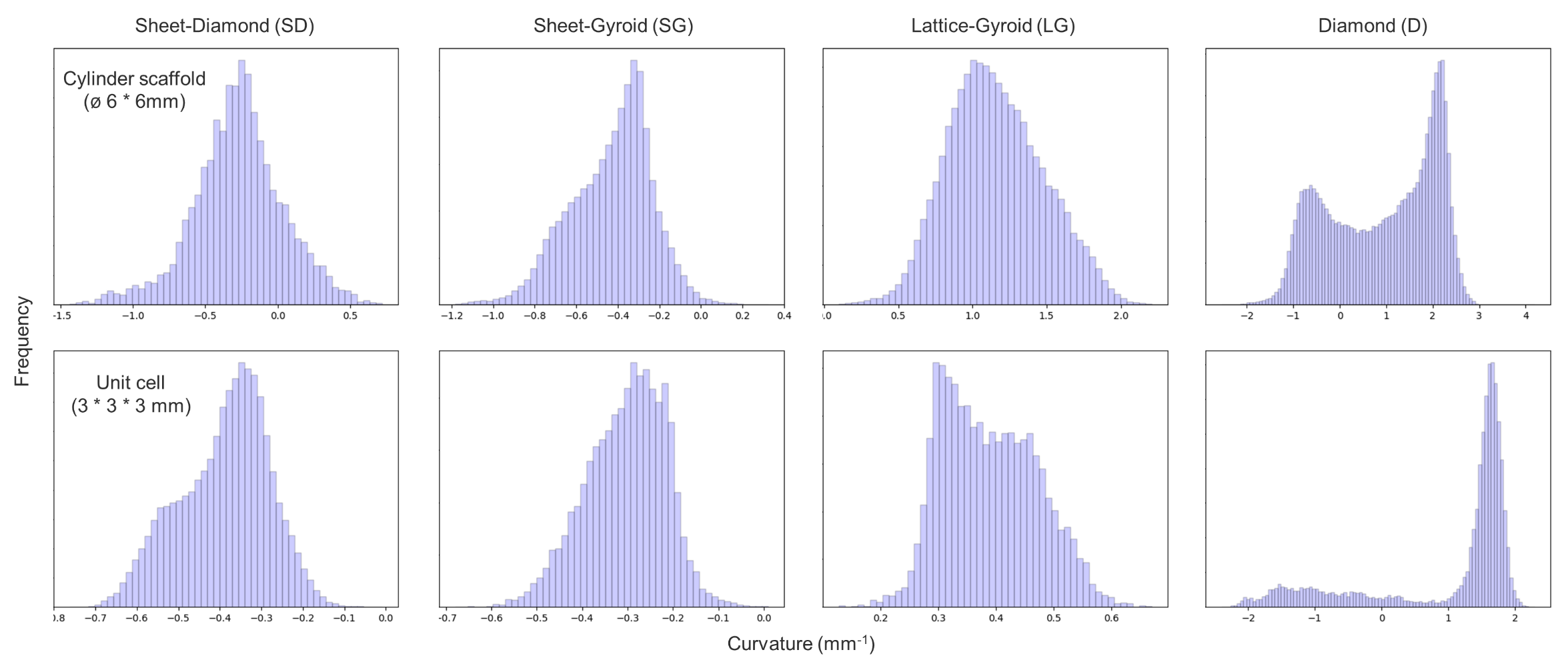


1. Mean curvature distribution of three TPMS and Diamond porous scaffolds.


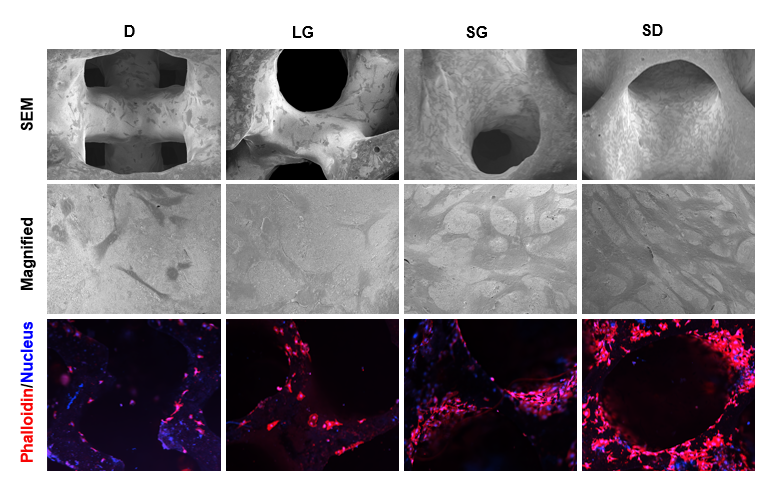


1. *In Vitro* cell adhesion of scaffolds with different structural units. All scaffolds are treated by high-temperature oxidation and hydrothermal processes.


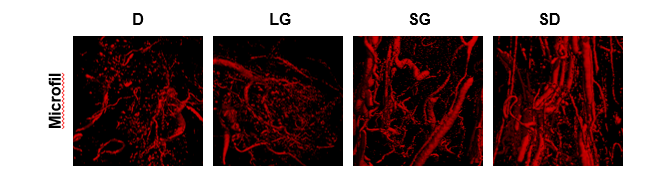


1. 3D reconstruction of neovascularization from Microfil angiography.


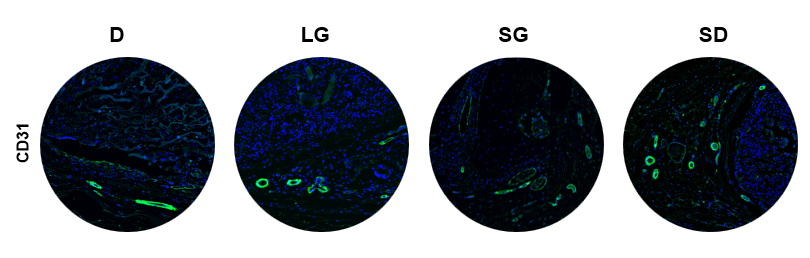


1. Immunostaining of CD31 in vivo.


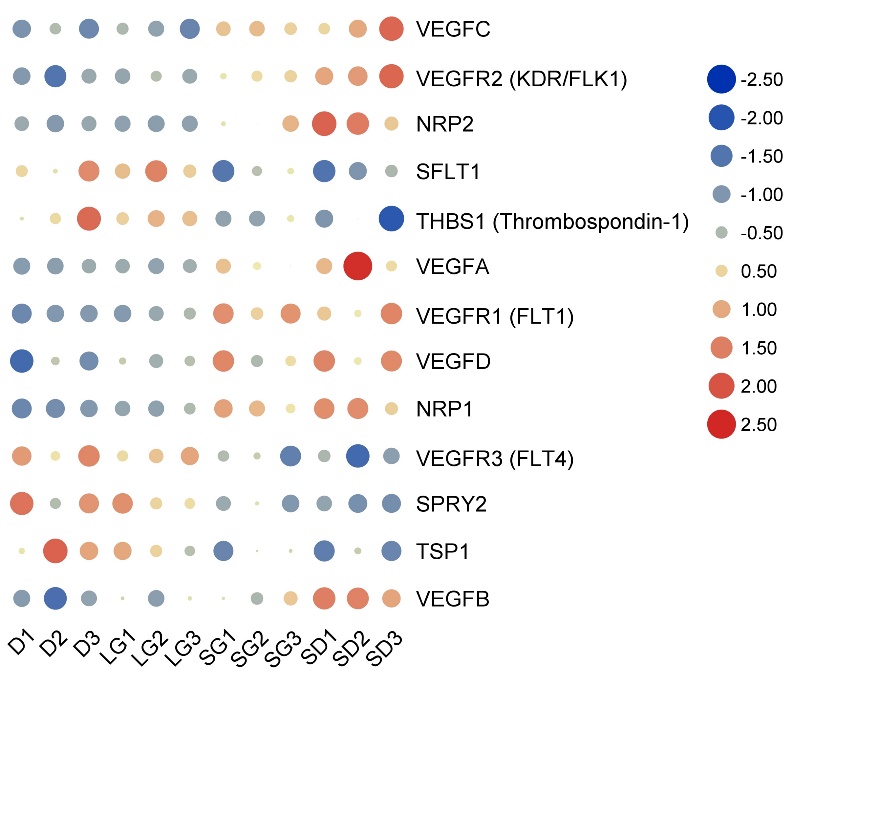


1. Heatmap of the impact of scaffolds of different structural units on key angiogenic gene expression


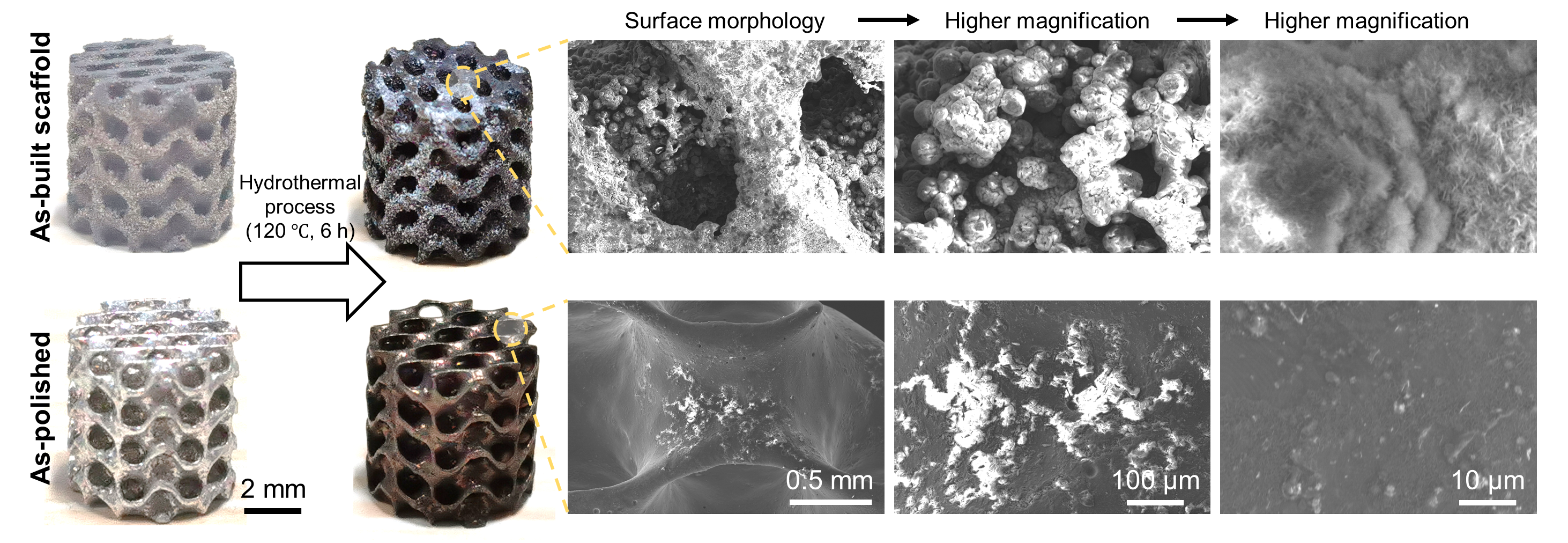


1. Image and surface morphologies of as-built and as-polished scaffolds with only hydrothermal treatment


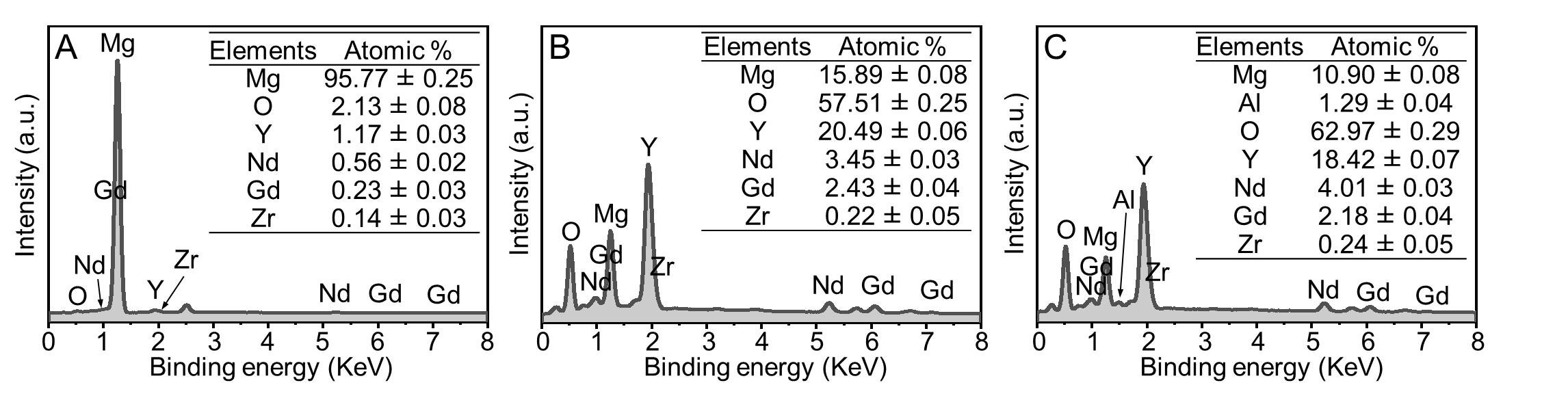


1. Energy dispersive spectroscopy (EDS) spectra of the scaffold surfaces: (**A**) AP, (**B**) HTO, and (**C**) HTO-LDH.


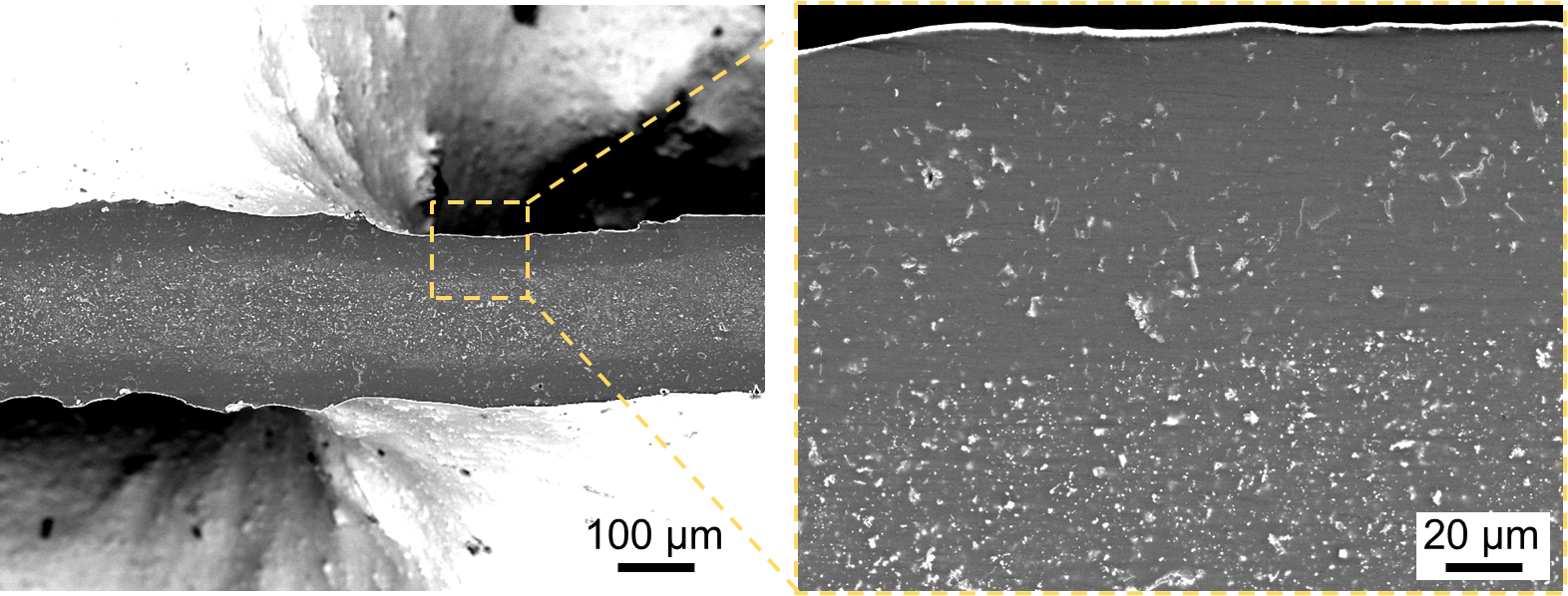


1. SEM backscattered electron images of HTO scaffold.


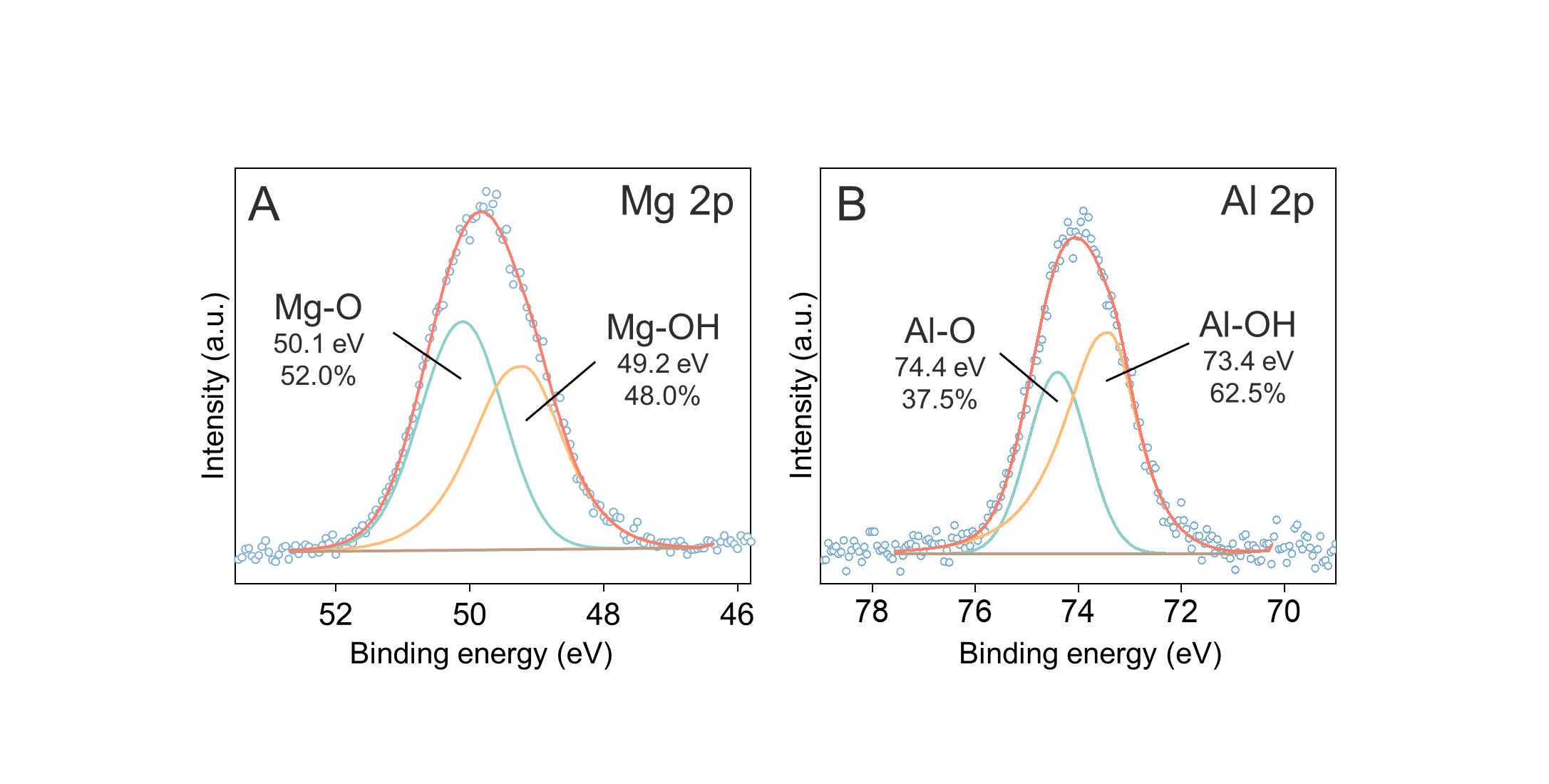


1. High-resolution X-ray photoelectron spectroscopy (XPS) spectra of HTO-LDH alloy. (**A**) Mg 2p and (**B**) Al 2p spectra.


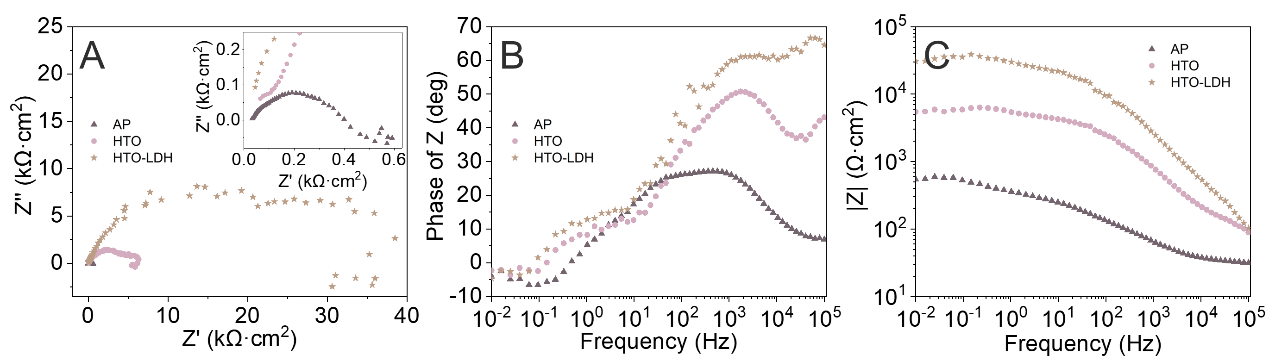


1. Electrochemical corrosion behavior. (**A**) Nyquist plots. (**B**-**C**) Bode plots of the phase angle and impedance modulus |Z| versus frequency.


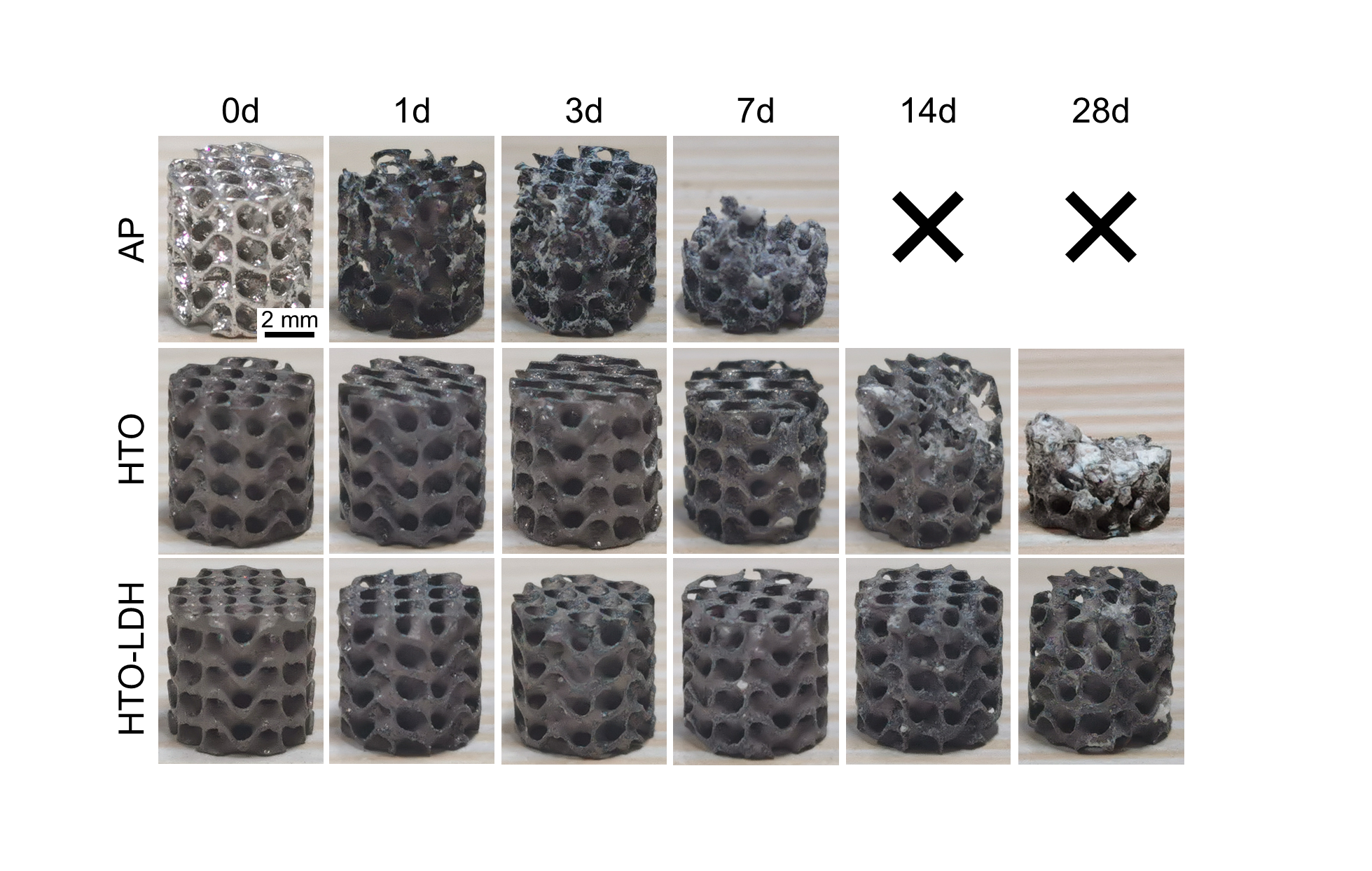


1. AP, HTO, and HTO-LDH scaffolds after 0, 1, 3, 7, 14, 28 days immersion in r-SBF solution at 37℃.


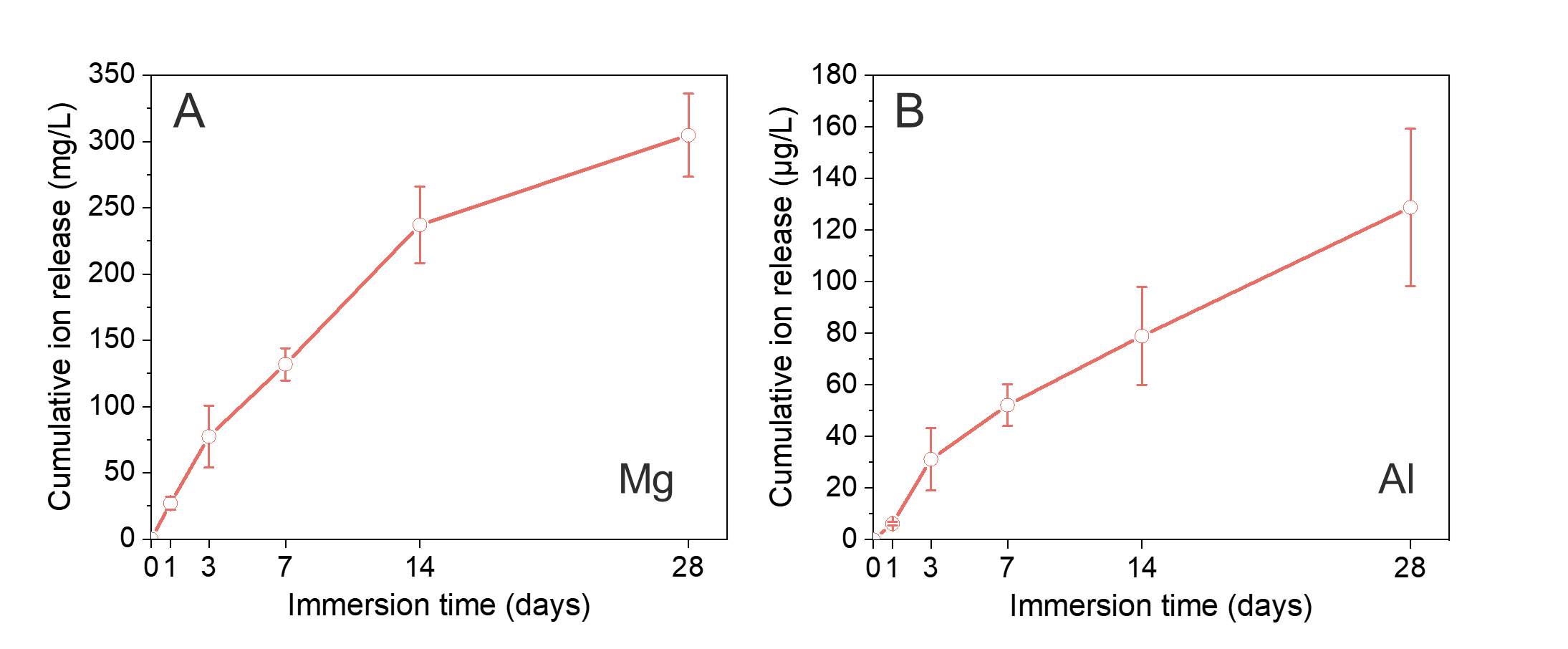


1. Figure R1. Cumulative release amount of Mg and Al ions in HTO-LDH scaffolds. Data are presented as mean values ± s.d. (*n* = 3). also added to Supplementary Information as Supplementary Figure xx

1. Figure R2. Al content in HTO-LDH scaffolds. Weight of HTO-LDH scaffolds in this study is 101 ± 1 mg. Data are presented as mean values ± s.d. (*n* = 3). Added to supplementary information as Supplementary Figure xx


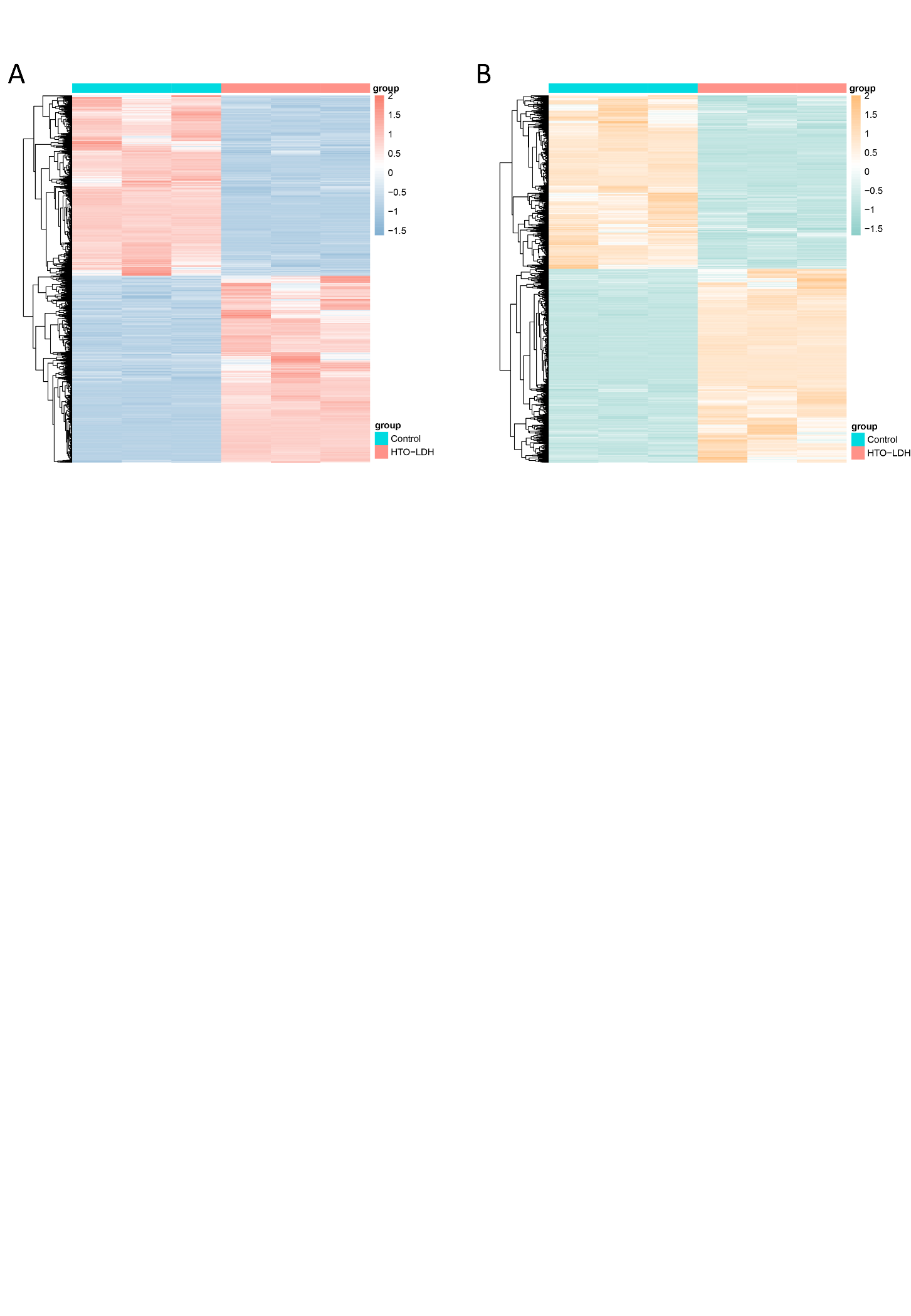


1. Heatmap of the differentially expressed genes identified by transcriptome sequencing: (**A**) hBMSCs, (**B**) HUVECs


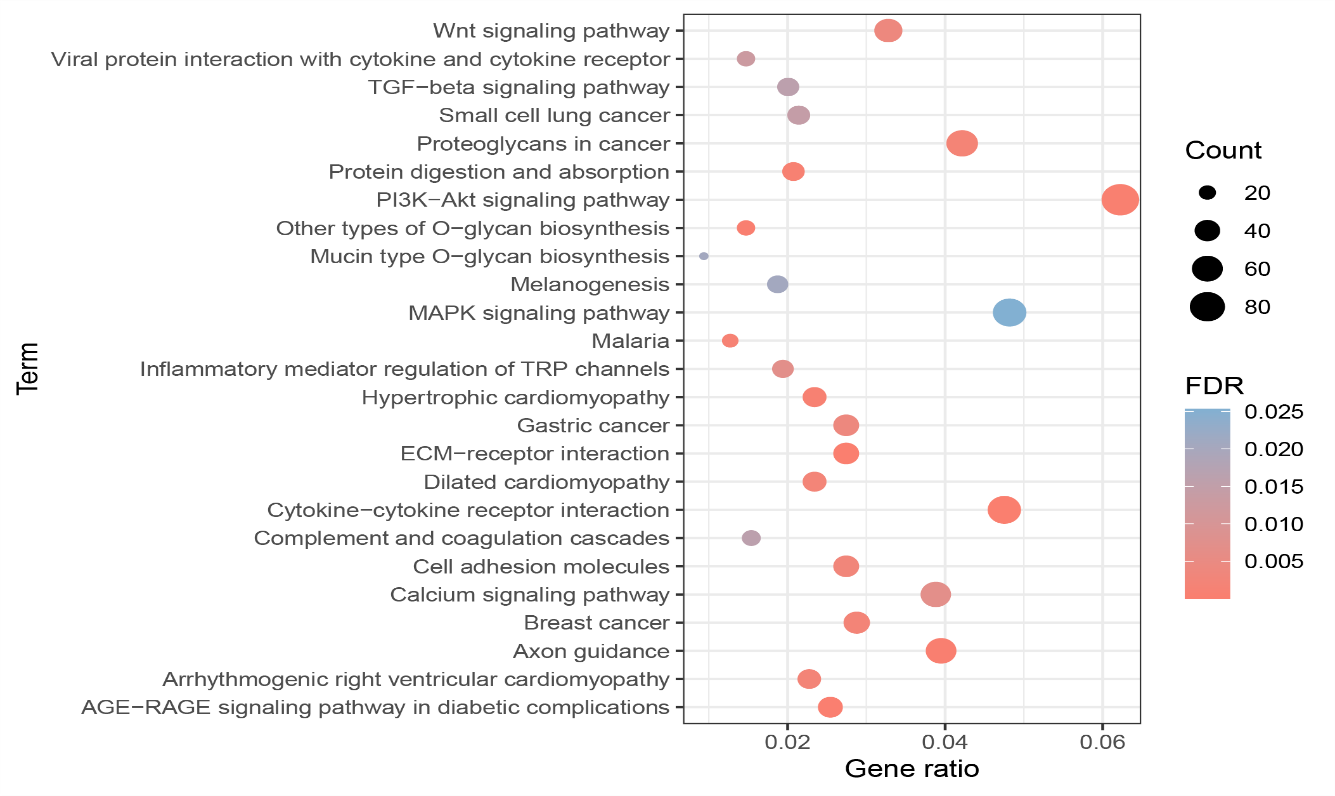


1. Top 20 signal pathways enriched by differentially expressed genes of hBMSCs using KEGG analysis.


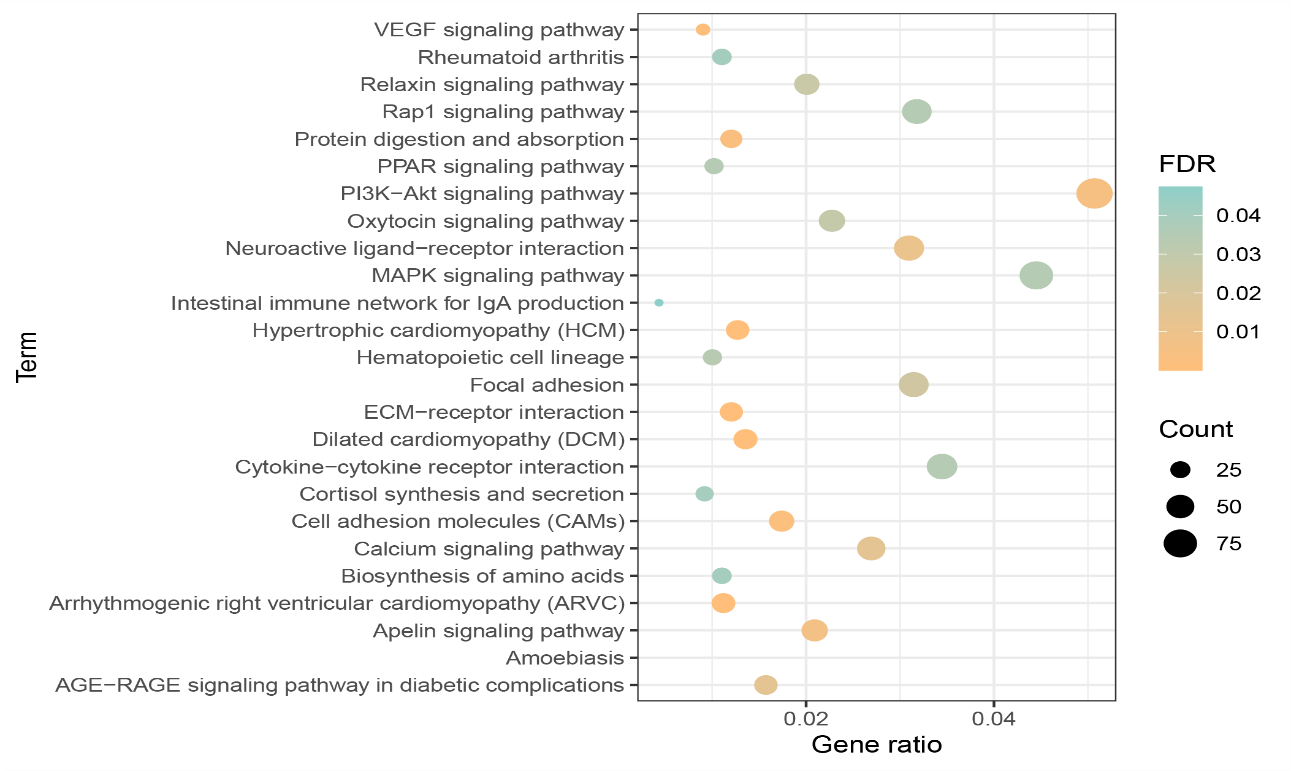


1. Top 20 signal pathways enriched by differentially expressed genes of HUVEC using KEGG analysis.


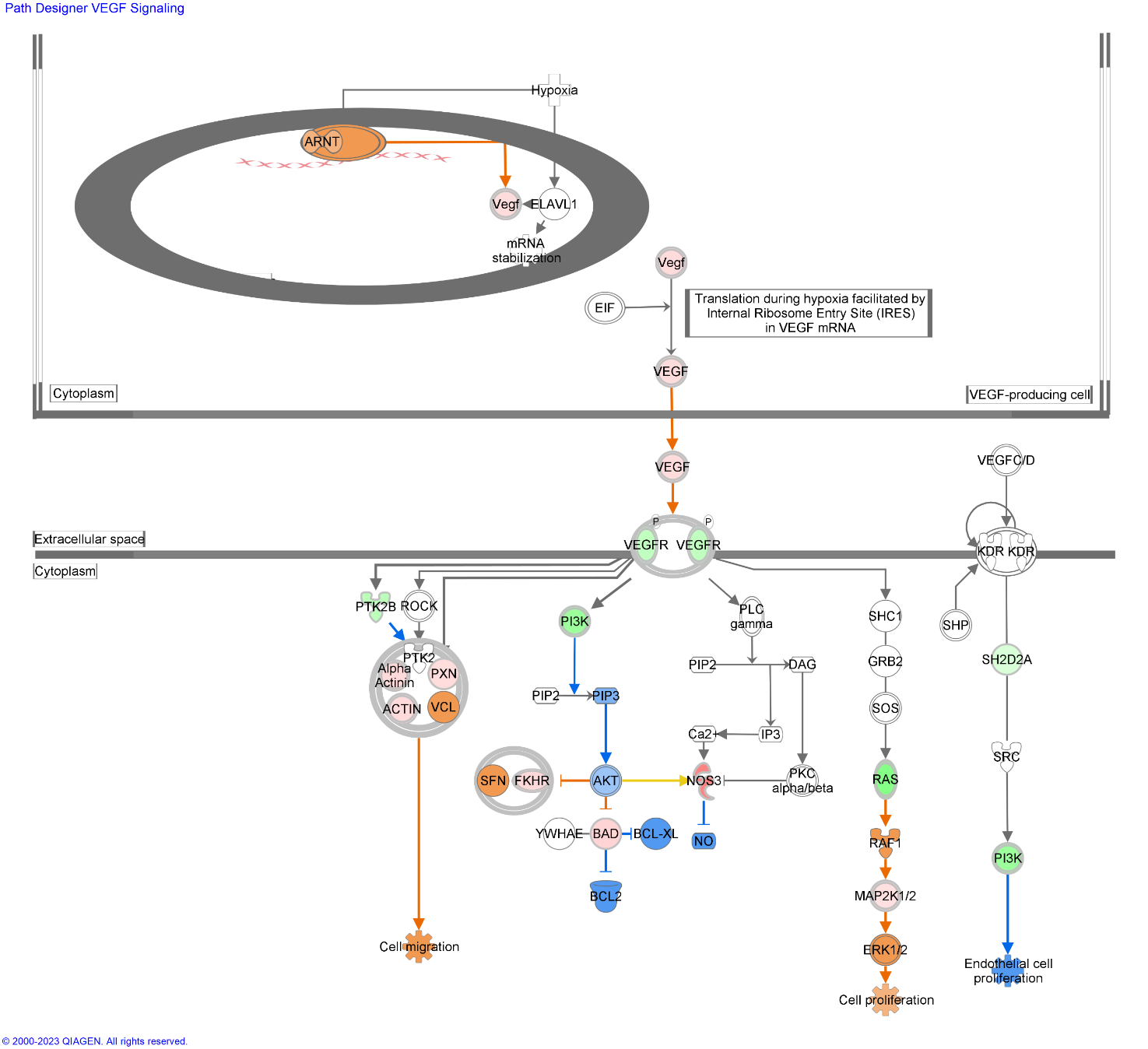


1. Differentially expressed genes enriched in the BMP signal pathway.


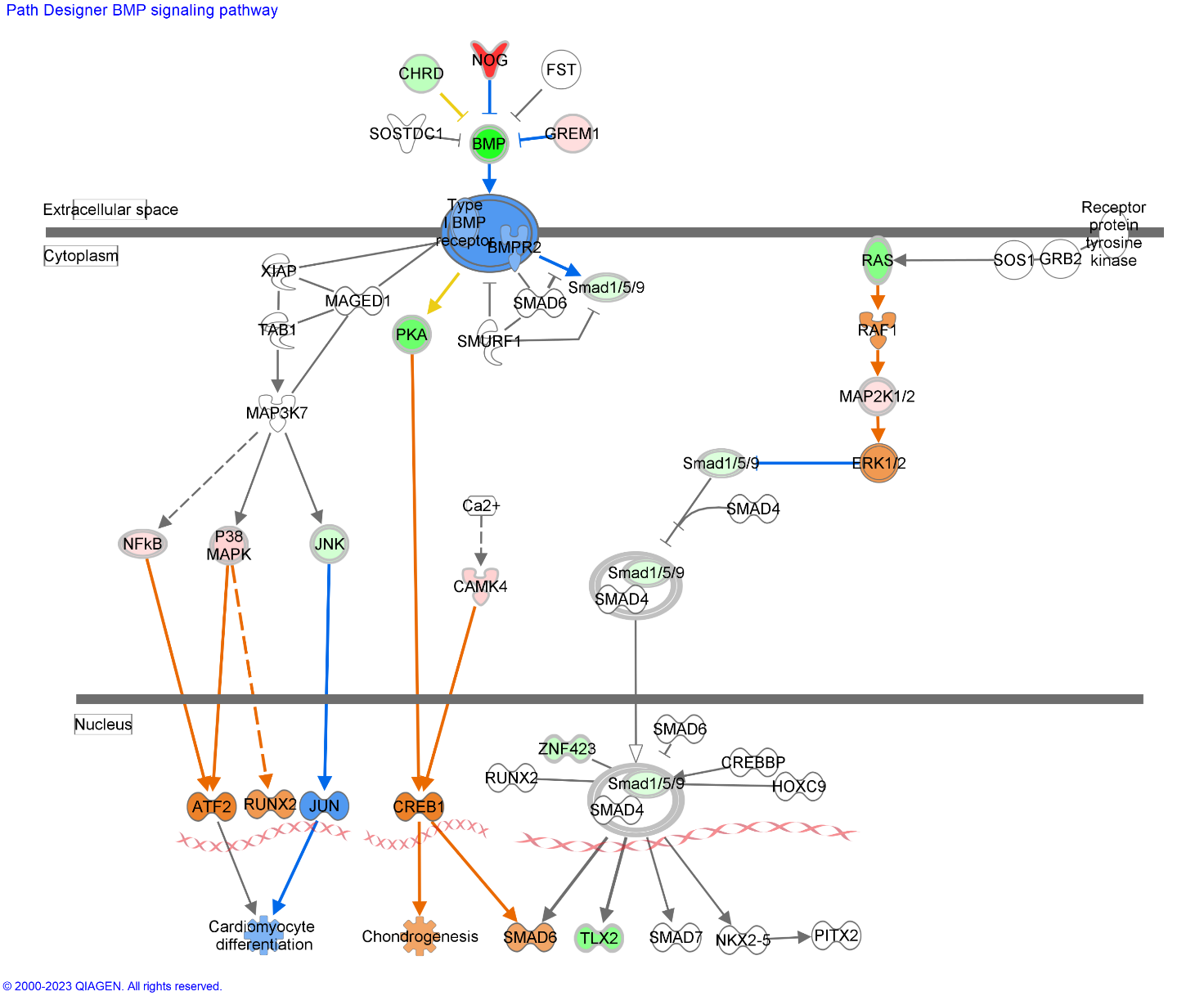


1. Differentially expressed genes enriched in the VEGF signal pathway.


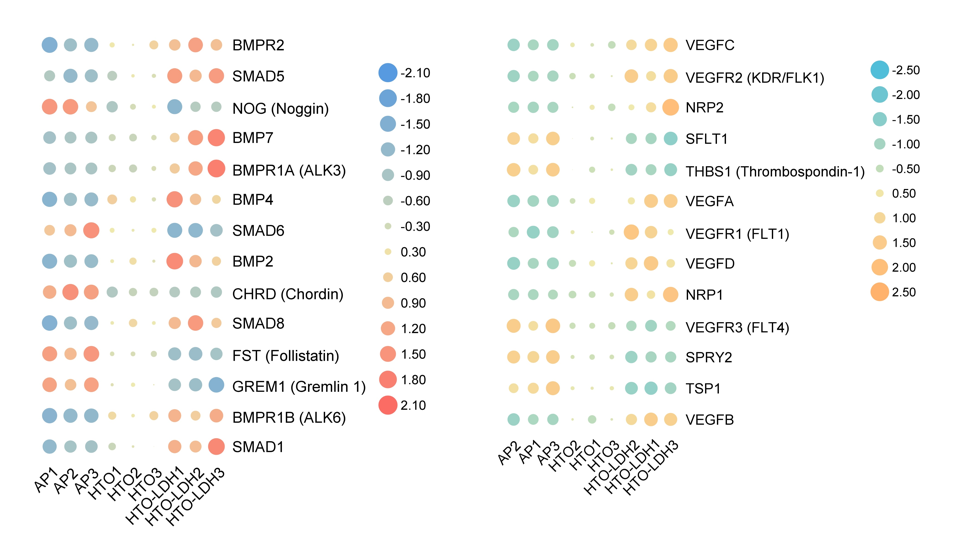


1. Heatmap of key gene expression in BMP and VEGF pathways *in vitro.*


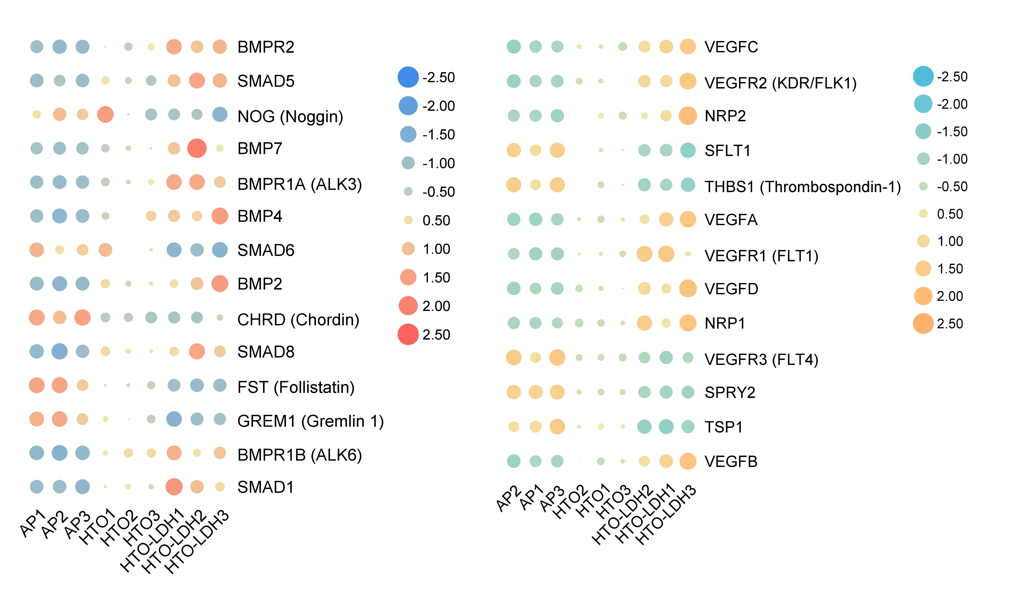


1. Heatmap of key gene expression in BMP and VEGF pathways *in vivo.*


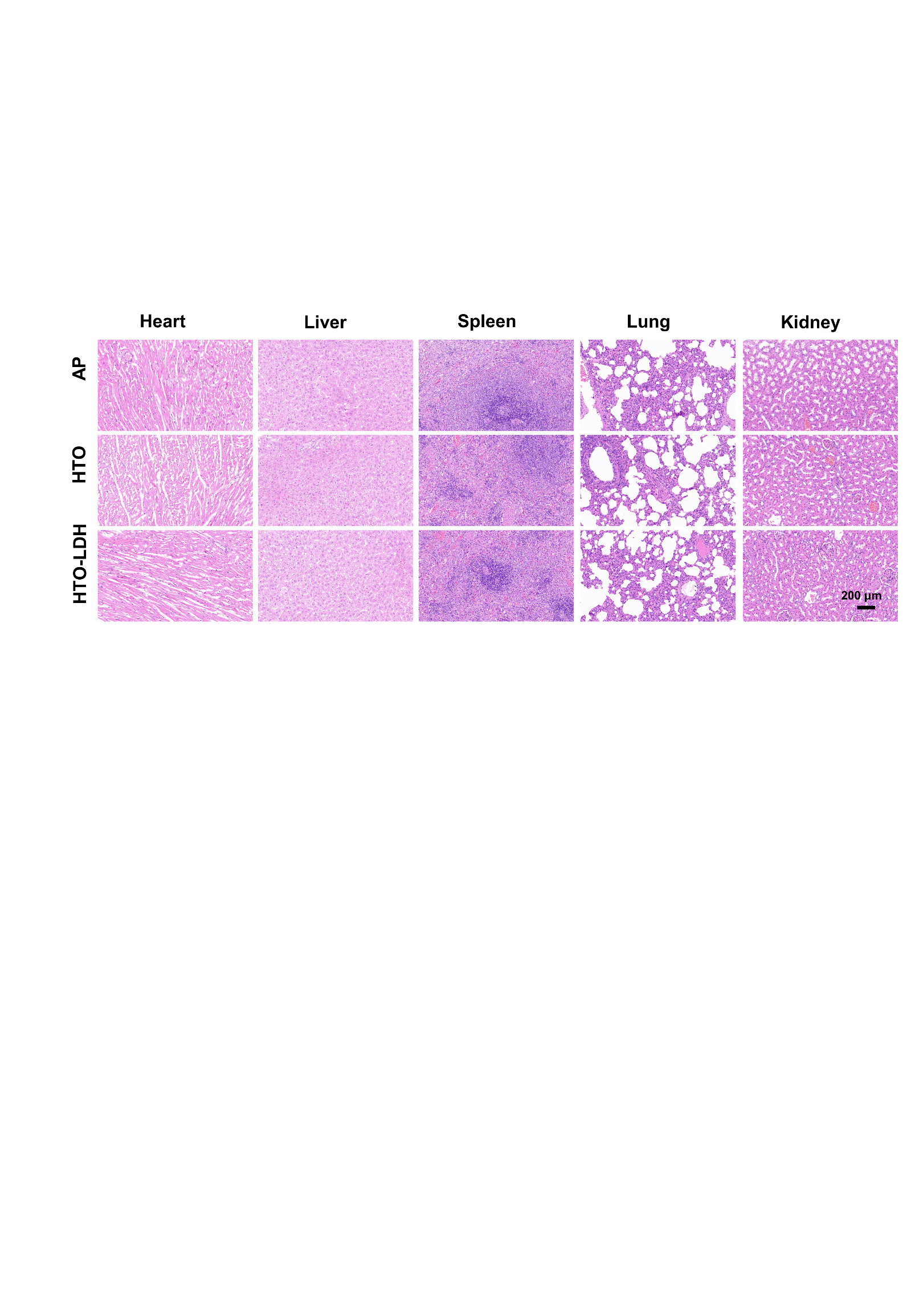


1. Tissue sections H&E staining of the heart, liver, spleen, lung, and kidney taken at 12 weeks after scaffold implantation.

**Table S1.** Mechanical properties from compression tests in Figure 2.

| Group | Yield strength (MPa) | Compressive strength (MPa) | Elastic modulus (MPa) |
| --- | --- | --- | --- |
| Sheet-Diamond | 32.4±2.7 | 48.8±6.0 | 1502±125 |
| Sheet-Gyroid | 29.0±2.2 | 46.1±3.0 | 1547±113 |
| Lattice-Gyroid | 20.7±1.6 | 32.9±1.2 | 1133±102 |
| Diamond | 22.3±0.3 | 35.0±1.9 | 1200±81 |

**Table S2.** Element ratios (at.%) measured by EDS in Figure 3.

| Position | Mg | O | Y | Nd | Gd | Zr | Al | Possible phases |
| --- | --- | --- | --- | --- | --- | --- | --- | --- |
| 001 | 97.10 | 0.96 | 1.03 | 0.54 | 0.22 | 0.15 | - | α-Mg |
| 002 | 84.28 | 8.95 | 5.25 | 0.57 | 0.45 | 0.50 | - | Y_2_O_3_ |
| 003 | 9.14 | 60.80 | 23.48 | 3.47 | 2.87 | 0.24 | - | Y_2_O_3_ |
| 004 | 17.19 | 60.84 | 11.13 | 8.21 | 2.50 | 0.13 | - | Nd_2_O_3_ |
| 005 | 10.89 | 60.37 | 19.55 | 5.05 | 2.70 | 0.26 | 1.18 | LDH |

**Table S3.** Mechanical properties from compression tests in Figure 4.

| Group | Yield strength (MPa) | Compressive strength (MPa) | Elastic modulus (MPa) |
| --- | --- | --- | --- |
| AP-0d | 32.4±2.7 | 48.8±6.0 | 1502±125 |
| AP-1d | 14.6±4.5 | 19.0±7.2 | 738±250 |
| AP-3d | 0.5±0.3 | 1.5±0.4 | 74±48 |
| HTO-0d | 20.5±0.4 | 37.5±1.0 | 1091±145 |
| HTO-1d | 18.7±1.4 | 32.2±1.2 | 778±187 |
| HTO-3d | 16.5±1.7 | 25.3±1.4 | 707±284 |
| HTO-7d | 11.5±2.2 | 19.5±4.3 | 573±189 |
| HTO-14d | 7.0±1.3 | 9.6±1.8 | 290±59 |
| HTO-LDH-0d | 21.2±1.5 | 38.5±0.2 | 1108±110 |
| HTO-LDH-1d | 20.9±1.4 | 35.0±2.2 | 934±232 |
| HTO-LDH-3d | 20.2±1.0 | 31.1±2.2 | 844±126 |
| HTO-LDH-7d | 18.4±1.4 | 31.2±5.0 | 779±284 |
| HTO-LDH-14d | 14.2±3.5 | 24.5±5.3 | 708±302 |
| HTO-LDH-28d | 12.2±4.2 | 24.9±7.6 | 597±149 |

**Table S4.** Osteogenic and angiogenic-related gene primer sequences used in the qPCR experiments.

| **Gene Name** | **Forward Sequences** | **Reverse Sequences** |
| --- | --- | --- |
| GAPDH | GTCTCCTCTGACTTCAACAGCG | ACCACCCTGTTGCTGTAGCCAA |
| ALP | GGTCAGGTTTCAACAGCCCTAG | GCTCATTCCGATTGTCGTGGAG |
| OCN | CGCTACCTGTATCAATGGCTGG | CTCCTGAAAGCCGATGTGGTCA |
| Runx2 | CCCAGTATGAGAGTAGGTGTCC | GGGTAAGACTGGTCATAGGACC |
| VEGF | TTGCCTTGCTGCTCTACCTCCA | GATGGCAGTAGCTGCGCTGATA |
| ANG1 | CAACAGTGTCCTTCAGAAGCAGC | CCAGCTTGATATACATCTGCACAG |
